# The role of the aging process and related factor *EMP1* in promoting progression of resectable pancreatic cancer

**DOI:** 10.1016/j.gendis.2024.101490

**Published:** 2024-12-15

**Authors:** Junfeng Zhang, Jianyou Gu, Tao Zhang, Renpei Xia, Jianbo Li, Mingda Tan, Yongjun Yang, Jifeng Xiang, Bin Xie, Rong Tang, Wangge Li, Xianxing Wang, Shixiang Guo, Huaizhi Wang

**Affiliations:** a University of Chinese Academy of Sciences (UCAS) Chongqing School, Chongqing Medical University, Chongqing 401147, China; b Institute of Hepatopancreatobiliary Surgery, Chongqing General Hospital, Chongqing University, Chongqing 401147, China; c Chongqing Key Laboratory of Intelligent Medicine Engineering for Hepatopancreatobiliary Diseases, Chongqing 401147, China; d Chongqing School, University of Chinese Academy of Sciences, Chongqing 400714, China; e Institute of Clinical Nutrition, Chongqing General Hospital, Chongqing University, Chongqing 401147, China; f Department of Clinical Laboratory, The First Affilitated Hospital of Chongqing Medical University, Chongqing 401147, China

**Keywords:** Aging, *EMP1*, Pancreatic cancer, *PI3K/AKT*, Postoperative prognosis

## Abstract

Pancreatic cancer (PC) is a highly malignant neoplasm of the digestive system. The primary objective of this investigation is to elucidate the intricate mechanisms underlying the role of the aging process and the related factor Epithelial membrane protein 1 (EMP1) in PC progression. We established a prognostic model pertinent to the aging process that could be applied in postoperative PC patients. *In vitro* assays were employed to elucidate the impact of *EMP1* on PC cell function. We employed lentiviral vectors for both knockdown and overexpression of *EMP1* in Panc02 cells, followed by the establishment of subcutaneous, pulmonary metastasis, and orthotopic pancreatic liver metastasis models in mice. Using tissue microarrays, we evaluated the expression of *EMP1* and its downstream entities, and then conducted clinical correlation analysis. A predictive Age-Related Score (ARS) system based on age-associated prognostic genes was developed, offering precise prognostic predictions for postoperative PC patients, which could be applied well at the single-cell level, showing diverse aging, epithelial–mesenchymal transition (EMT), cell migration, cell proliferation, and *PI3K/AKT* signaling activity in high and low ARS risk cells. *EMP1* was identified as a pivotal molecule in the ARS system and is associated with poor prognosis. Besides, *EMP1* could enhance the proliferation, migration, and invasion of PC cells both *in vitro* and *in vivo* by augmenting the *PI3K/AKT* signaling cascade. In essence, this research formulated an aging-centric prognostic model for postoperative PC and pinpointed *EMP1* as an oncogenic factor facilitating tumor cell EMT during the aging trajectory in resectable PC patients.

## Introduction

Pancreatic cancer (PC) is a formidable challenge in oncology due to its aggressive nature and dismal prognosis, necessitating ongoing research into effective interventions.^1^, ^2^, ^3^ Pancreatoduodenectomy has emerged as the most effective surgical option for PC, offering a potential cure in select cases.^4^ There is a significant disparity in the prognosis between patients who are candidates for curative resectionand those who are not. However, a focused evaluation of outcomesfollowing curative resection in PC patients remains unexplored. Aging, with its associated physiological and molecular changes, affects the immune system and tissue structure, possibly enhancing the propensity for tumor spread.^5^, ^6^, ^7^, ^8^, ^9^, ^10^, ^11^, ^12^, ^13^ These changes, including weakened immune responses, “inflammaging”, and alterations in tissue architecture, contribute to the tumor’s ability to metastasize and evade treatment.^7^, ^8^, ^9^, ^10^, ^11^ Furthermore, cellular senescence complicates the tumor microenvironment by promoting a pro-inflammatory state that promotes tumor growth.^12^^,^^13^

Clinical evidence underscores the importance of age in the metastatic potential of PC, linking age-related changes to increased tumor invasion and metastasis.^14^^,^^15^ At the molecular level, aging influences key signaling pathways, potentially enhancing the metastatic capabilities of tumor cells and leading to genetic and epigenetic alterations that favor aggressive tumor behavior.^6^^,^^16^, ^17^, ^18^

This study focuses on epithelial membrane protein 1 (EMP1), a transmembrane glycoprotein implicated in various aggressive cancers.^19^, ^20^, ^21^, ^22^, ^23^, ^24^, ^25^, ^26^ We investigate *EMP1*’s role in PC metastasis and its potential as a diagnostic and prognostic biomarker. Utilizing data from the Surveillance, Epidemiology, and End Results (SEER) database and The Cancer Genome Atlas (TCGA), we developed a risk-prognostic model based on age-related prognostic genes, highlighting *EMP1*’s provital role. Our findings, derived from clinical tissue microarrays, single-cell and bulk sequencing, and mechanistic experiments, suggest *EMP1* is involved in PC metastasis via the *PI3K/AKT* signaling pathway, offering insights into potential novel therapeutic strategies and personalized treatment for PC patients.

## Materials and methods

### Data acquisition and processing

**Figure 1:**
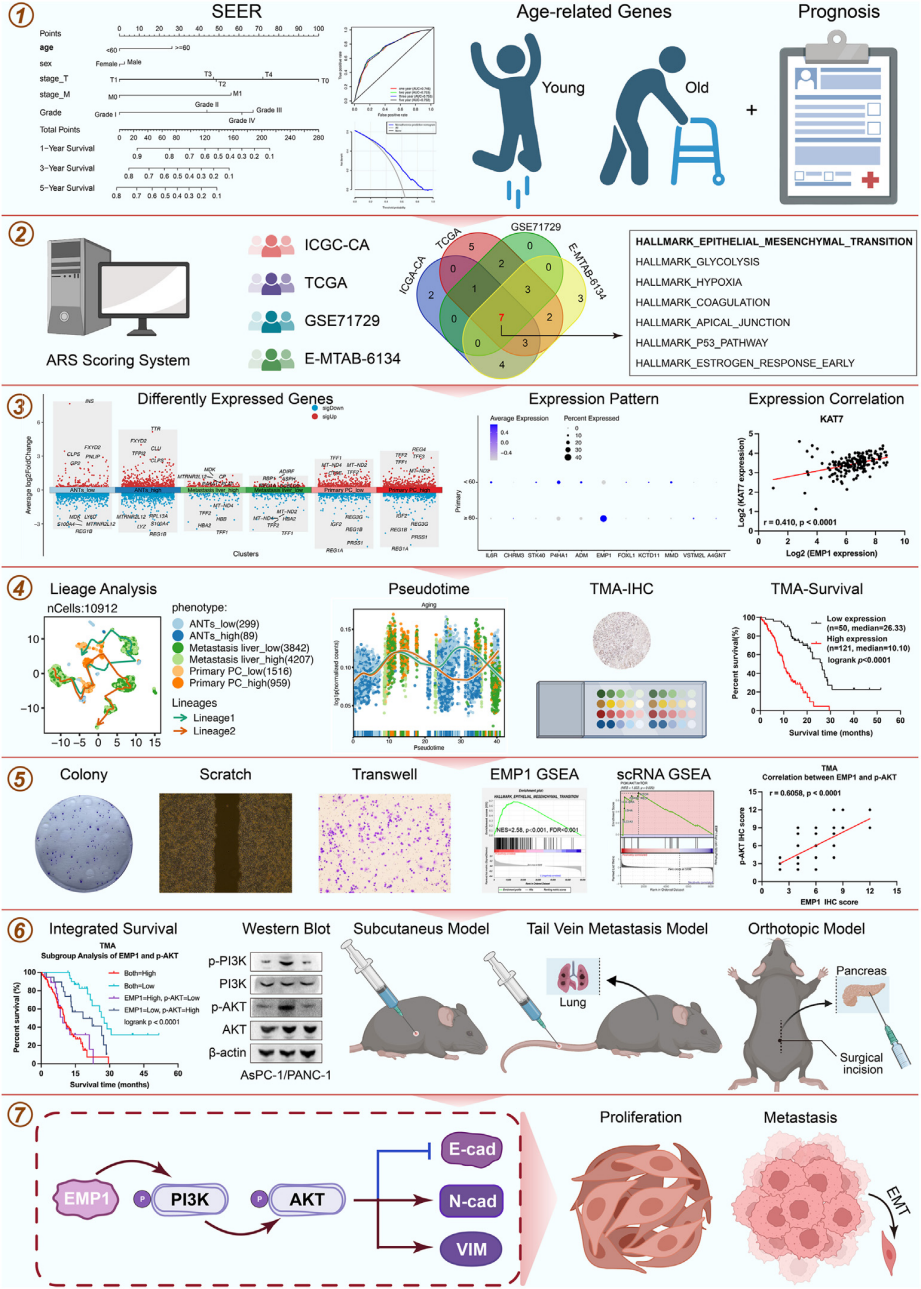
The schematic diagram of the study. A prognostic framework for pancreatic cancer (PC) derived from the Surveillance, Epidemiology, and End Results (SEER) database was established. A gene expression-based prognostic model, pertinent to aging-associated resectable PC, was established and applied at the single-cell resolution. *In vitro* and *in vivo* assays were employed to elucidate the impact and underlying mechanisms of *EMP1* on PC cell proliferation, invasion, and migration. We used tissue microarrays from PC patients to evaluate the expression of *EMP1* and its downstream entities via immunohistochemistry, followed by clinical correlation analysis.

The research overview is shown in Figure 1. Data for 18,902 PC patients diagnosed between 2010 and 2015 were sourced from the SEER database, which was publicly released in April 2018. This database comprises clinical data of PC patients such as age, sex, grade, T stage, N stage, M stage, survival duration, and survival status.^27^ Gene expression profiles for PC were retrieved from several databases: EBI ArrayExpress (E-MTAB-6134), Gene Expression Omnibus (GEO, GSE62165, and GSE71729), International Cancer Genome Consortium (ICGC, PACA-CA), The Cancer Genome Atlas (TCGA-PAAD), and Genotype-Tissue Expression. For survival analysis, cases with incomplete prognostic information were systematically excluded from the respective datasets. The ICGC-CA dataset, comprising 182 PC patients, served as the training cohort. Three additional independent datasets, namely TCGA (*n* = 177), GSE71729 (*n* = 125), and E-MTAB-6134 (*n* = 288), were designated as validation cohorts. Besides, we used GSE155698, GSE154778, and GSE156405 single-cell RNA-sequencing (scRNA-seq) datasets to analyze primary PC, metastasis PC, and adjacent normal tissue (ANT) cells at the single-cell level.

### Establishment and validation of the ARS system

Initially, the TCGA-PAAD dataset was stratified into two groups based on the age threshold of 60 years: the elderly cohort and the younger cohort. Differential gene expression analysis was conducted using the ‘limma’ package, identifying 397 age-related differentially expressed genes with criteria set at logFC = 0.263 and FDR < 0.05. These 397 age-related genes were then subjected to univariate Cox regression analysis to identify prognostic genes associated with metastatic capability within the training cohort (ICGC-CA) using the ‘survival’ package (*p* < 0.01 threshold). Subsequently, a multivariate Cox regression analysis was employed to establish the ARS system. The area under the receiver operating characteristic (ROC) curve was computed to assess the predictive accuracy of the model. Calibration plots were generated to evaluate the model’s precision. Harrell’s C-index was calculated to determine the model’s discriminative ability. Bias-corrected C-index values were derived through bootstrap validation with 1000 bootstrap samples. Decision curve analysis was executed to quantify the net benefit of the model at various threshold probabilities within the cohort, providing insights into itsclinical utility.

### Single-cell analysis process

The scRNA-seq data was processed utilizing the Seurat package. Tumor cells from peri-tumoral, primary, and hepatic metastatic PC tissues were extracted. Cells with detected gene counts exceeding 6000 or below 200, and those with mitochondrial gene proportions surpassing 10% in individual cells were filtered out. Following normalization, identification of highly variable genes, and principal component analysis, uniform manifold approximation and projection dimensionality reduction was executed for visualization. Differential gene expression across cell clusters was identified using the FindAllMarkers function. Visualization of differential genes for each cluster was achieved using the scRNAtoolVis or SCP package.

### Cell scoring and enrichment analysis of various characteristics

In a novel approach, this study adapted the ARS scoring system derived from bulk RNA-seq to the single-cell level. Utilizing the predict function from the glmnet package, the ARS scoring system was applied to individual cells, yielding ARS scores for each. The AUCell function was employed to compute scores for cellular features such as aging, epithelial–mesenchymal transition (EMT), cell migration, cell proliferation, *PI3K/AKT*/*mTOR*, *PI3K/AKT* activation, and *PI3K/AKT* signaling. Gene ontology (GO), Kyoto Encyclopedia of Genes and Genomes, and Gene Set Enrichment Analysis (GSEA) analyses and visualizations for different cell clusters were conducted using the clusterProfiler and GseaVis packages.

### Cell trajectory analysis

Cell trajectory analysis was employed to infer the progression of tumor cells, and cell lineage was identified based on single-cell RNA expression characteristics. The RunSlingshot and RunDynamicFeatures functions from the SCP package were utilized for cell trajectory inference and dynamic feature analysis.

### Cell lines and reagents

The AsPC-1, PANC-1, and Panc02 cell lines were obtained from the Shanghai Institute of Biochemistry and Cell Biology and Zhong Qiao Xin Zhou Biotechnology Co., Ltd. Cells were cultured in RPMI 1640 medium, enriched with 10% fetal bovine serum and 1% antibiotics, and incubated at 37 °C in a 5% CO_2_ atmosphere. The PI3K inhibitor LY294002 (10 μM, MCE, USA) was used to treat cells for 24 h.

### Western blot analysis

Standard Western blotting protocols, as previously described,^28^ were utilized, employing specific antibodies targeting EMP1, E-cadherin, N-cadherin, VIM, p-PI3K, PI3K, p-AKT, AKT (1:1000) and β-actin (1:5000; Cell Signaling Technology, USA). Secondary antibodies were horseradish peroxidase-conjugated. Protein levels were normalized against β-actin.

### Tissue microarrays (TMA) and immunohistochemical analysis (IHC)

Ethical clearance was granted by the Chongqing General Hospital Ethics Committee, with the approval number KY S2002-079-01. Tissue samples were optained from the Hepatopancreatobiliary Surgery of Chongqing General Hospital. All patients have signed informed consent forms. Tissue samples with complete clinical information were collected. IHC was executed on two TMAs, encompassing 171 diagnosed PC cases and 71 normal pancreatic specimens. For both human and animal tissue samples, standard protocols (Maixin, Fuzhou, China) were employed to perform IHC and hematoxylin-eosin staining on formalin-fixed, paraffin-embedded sections.^29^ Histological evaluations of paraffin sections from animal experiments utilized primary antibodies against EMP-1 (1:500, Servicebio), Ki-67 (1:1000, Servicebio), E-cadherin (1:500, Servicebio), N-cadherin (1:500, Proteintech), VIM (1:2000, Servicebio), p-PI3K (1:200, ThermoFisher), and p-AKT (1:200, CST). The IHC scoring combined staining intensity with the proportion of stained cells. The intensity of staining was assessed and scored as follows: 0 for no staining, 1 for faint staining, 2 for medium staining, and 3 for intense staining. The proportion of cells exhibiting positive staining was categorized and scored as follows: 0 for less than 5%, 1 for 5%–25%, 2 for 26%–50%, 3 for 51%–75%, and 4 for more than 75%. The final staining index was calculated by multiplying these two scores, and samples were subsequently categorized based on their staining index values.

### Gene manipulation techniques

Human *EMP1*-specific shRNA was obtained from RiboBio Co. (Guangzhou, China). To overexpress human *EMP1*, the entire gene sequence was integrated into the pEX-3 (pGCMV/MCS/Neo) vector (GenePharma, Shanghai, China). In related animal experiments, Panc02 cells were transfected using LentiCRISPRv2 vectors harboring sgRNA targeting a specific genomic locus. The sgRNA specific to mouse *EMP1* was acquired from Tsingke Co. (Beijing, China). To elevate mice *EMP1* expression, the complete gene was inserted into the PLVX-puro vector (Tsingke, Beijing, China). Transfections were executed using Lipofectamine 3000 (Invitrogen, Carlsbad, USA), adhering to the manufacturer’s recommendations.

### Colony formation assay

After transfection, cells were seeded in 6-well plates at a density of 10^3^/well. Post a 14-day culture, the cells were fixed using 4% paraformaldehyde and subsequently stained with crystal violet (Beyotime, China).

### Migration and invasion assays

For the wound healing assay, cells were seeded into 6-well plates after transfection. Controlled scratches were made in the cell layer using a sterile 10 μL micropipette tip. Images capturing the wound closure were recorded immediately and again after 24 h. For the transwell migration assay, cell culture inserts featuring 8 μm pore-sized transparent PET membranes (Corning, MA, USA) were employed. Cells, at a density of approximately 5 × 10^4^, were suspended in a serum-free medium and added to the upper chamber. The lower chamber received medium supplemented with 10% FBS. After 10–12 h of incubation, migrating cells were fixed with 4% paraformaldehyde, stained using crystal violet (Beyotime, China), and quantified via ImageJ software. The invasion assay utilized Matrigel Invasion Chambers (Merck Millipore, USA) and followed a similar protocol to the migration assay.

### Animal experiments

Our work has been reported in accordance with the ARRIVE guidelines.^30^ All *in vivo* protocols were approved by the Ethics Committee of Chongqing General Hospital. Female C57BL/6J mice, aged between 4 and 6 weeks, were obtained from SpePharm Biotechnology Co. (Beijing, China), and randomized using a random number method. To elucidate the physiological effects of *EMP1 in vivo*, we employed lentiviral vectors for both knockdown and overexpression of *EMP1* in Panc02 cells, subsequently establishing subcutaneous, pulmonary metastasis, and orthotopic pancreatic liver metastasis models in C57BL/6J mice, with a total sample size of 70. Control and *EMP1*-suppressed groups were established for subcutaneous (7 *vs.* 7), pulmonary metastasis (7 *vs.* 7), and orthotopic pancreatic liver metastasis models (7 *vs.* 7). Additionally, vector with DMSO group (*n* = 7), overexpression of *EMP1* with DMSO group (*n* = 7), *PI3K/AKT* pathway antagonist group (*n* = 7), and overexpression of *EMP1* with *PI3K/AKT* pathway antagonist group (*n* = 7) were established for subcutaneous tumors. The sample size was design to achieve statistical significance. For the subcutaneous tumor model, 1 × 10^6^ Panc02 cells suspended in 100 μL of PBS were subcutaneously inoculated into the mice’s abdominal flank. Once palpable tumors were evident, mice underwent intraperitoneal treatments with PI3K inhibitor LY294002 (50 mg/kg) at three-day intervals. Tumor dimensions, including length (L) and width (W), were measured weekly with precision calipers, with the tumor volume deduced using equation (L × W^2^)/2. Systematic weekly evaluations were conducted to monitorthe weight and tumor growth. Four weeks after the initiation of the subcutaneous tumor model, mice were humanely sacrificed to ascertain tumor mass. For the orthotopic pancreatic tumor model, a central incision was made in the anterior abdominal wall, followed by the direct injection of 1 × 10^6^ cells into the distal pancreatic tissue. In the tail vein pulmonary metastasis model, 5 × 10^5^ tumor cells were injected into the mouse tail vein. Anesthesia was administered as required, using either isoflurane or pentobarbital sodium. Six weeks post-cellular injection, pancreatic, hepatic, and pulmonary tissue specimens were harvested to assess the metastatic potential of tumor cells under various therapeutic conditions. To control confounding factors, every data point was evaluated by three technicians, and the mean values were used.

### Statistical analysis and visualization

We employed GraphPad Prism (version 9.0) and R version 4.2.2 for our analyses and visualization. Results are expressed as the mean ± standard deviation (SD) derived from three distinct experiments. For comparisons involving two groups, the Student’s *t*-test was applied, while for multiple group comparisons, ANOVA followed by Tukey’s post-hoc test was used. This study incorporated a range of statistical methods, such as the Chi-square and log-rank tests. To pinpoint independent predictors of prognosis, the Cox proportional hazards regression model was adopted. For assessing the association between gene expression and pathway scores, we turned to the ssGSEA approach and Spearman’s correlation, facilitated by the GSVA package in R. All tests were two-sided, with a *p*-value threshold of less than 0.05 indicating statistical significance. The schematic was generated using the BioRender tool.

## Results

### Prognostic framework for PC derived from the SEER database

As delineated in Table S1, older PC patients were more likely to present with higher T stage, lower N stage, and worse differentiation. As outlined in Table S2, our initial approach involved a univariate Cox proportional hazards evaluation to discern elements potentially impacting PC prognosis. Advancing our analysis, factors with a *p*-value less than 0.05 in the univariate Cox assessment were integrated into a multivariate regression framework. This in-depth analysis identified age, gender, T stage, N stage, M stage, and differentiation as distinct determinants of PC prognosis. A notable finding from our analysis indicated that elderly PC patients exhibited a markedly poorer prognosis than their younger counterparts (HR = 1.531, *p* < 0.001).

### Nomogram for predicting overall survival (OS) outcomes

To provide clinicians with a more intuitive tool for evaluating patient outcomes, we devised a nomogram predicting OS based on the SEER dataset (Fig. S1A). The C-index, employed to predict OS, was recorded at 0.709. The ROC curve of this nomogram yielded area under curve scores of 0.746, 0.753, 0.755, and 0.752 for the 1-year, 2-year, 3-year, and 5-year forecasts, respectively (Fig. S1B). As illustrated in Fig. S1C, the decision curve analysis of the nomogram indicated a threshold probability range of 11%–89%. The 1-year, 2-year, 3-year, and 5-year survival calibration plots (Fig. S1D) showcased a strong alignment between the predicted outcomes from the nomogram and the actual recorded data.

### Establishment and validation of the ARS

**Figure 2:**
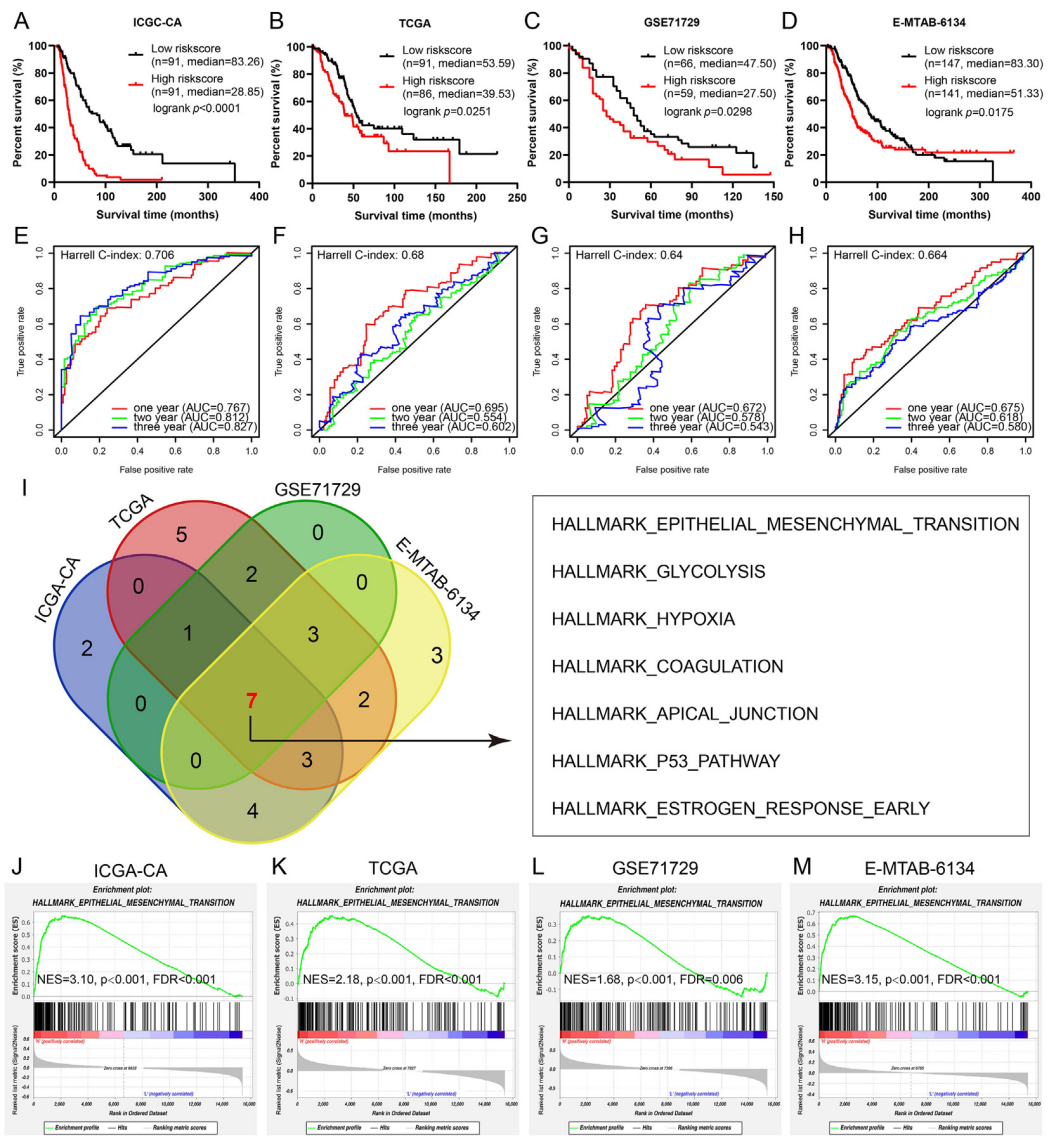
Identification and validation of age-related score. **(A**–**D)** Kaplan–Meier survival analyses for overall survival among pancreatic cancer (PC) patients are depicted, with data sourced from the ICGC-CA training cohort (A), The Cancer Genome Atlas test cohort (B), GSE71729 test cohort (C), and E-MTAB-6134 test cohort (D). Each analysis is categorized based on associated risk scores. **(E**–**H)** Time-dependent receiver operating characteristic curves for the prognostic model in the training and validation datasets. **(I)** A Venn representation illustrates overlapping significant pathways from gene set enrichment analysis (GSEA) between high and low-risk groups across both training and validation datasets. **(J**–**M)** GSEA analysis indicates a notable correlation between model scores and EMT in PC.

Gene expression patterns varied with age. To further delineate age-associated prognostic genes, we employed both univariate and multivariate Cox regression models, culminating in the construction of the ARS system. The ARS system included 11 genes: *IL6R, CHRM3, STK40, P4HA1, ADM, EMP1, FOXL1, KCTD11, MMD, VSTM2L,* and *A4GNT*. The ICGC-CA dataset served as our training set, while TCGA, GSE71729, and E-MTAB-6134 were utilized as independent validation sets. The data revealded a consistent trend in both training and validation sets: as risk scores escalated, the survival duration of all PC patients diminished (Fig. 2A–D). Figure 2E–H delineated the temporal evolution of the ROC curves for the prognostic model across the training and validation datasets. The Harrell C-index for the training set, ICGC-CA, was 0.706, whereas the indices for the validation sets TCGA, GSE71729, and E-MTAB-6134 were 0.68, 0.64, and 0.664, respectively. Calibration curves and decision curve analyses for both the training and validation cohorts were depicted in Figure S2A–D.

In summary, we have developed a predictive model based on age-associated prognostic genes, offering precise prognostic predictions for resectable PC patients. Comprehensive evaluations of the model, encompassing time-dependent ROC curves, the C-index, calibration plots, and decision curve analysis, further underscore its accuracy and clinical utility.

### Application of ARS score system at the single-cell level

We conducted a GSEA functional enrichment analysis based on the model’s scoring stratification using both the training and validation sets. As depicted in Figure 2I–M, the scoring system, based on age-associated genes, demonstrated a pronounced positive correlation with the EMT process. In this study, we innovatively applied the ARS scoring system, derived from bulk RNA-seq, to scRNA-seq data of PC to assess its efficacy at the single-cell level. We used uniform manifold approximation and projection for dimensionality reduction, and analyzed scRNA-seq data from primary PC, hepatic metastatic lesions, and adjacent normal tissues, resulting in 11 distinct cell clusters (Fig. S3A). By calculating the AUCell scores for ARS in each cell, we observed notably higher ARS scores in primary PC and hepatic metastatic lesionsthan in adjacent normal tissues (Fig. S3B). A low but significant correlation (*r* = 0.11, *p* < 0.001) suggested potential associations between ARS scores in hepatic metastatic lesions, rather than ANTs and primary PC with aging (Fig. S3C) and EMT (Fig. S3D). Cells were then classified into high and low-risk groups based on ARS scores, and differential genes for each group were identified (Fig. S3E). The marker for ANT with low ARS score was *INS*, while *TTR* served as the marker for ANTs with high ARS score. For metastasis to the liver with low ARS score, *ADIRF* was identified as the marker, and *MDK* was the marker for liver metastasis with high ARS score. In the context of primary pancreatic cancer, *TFF1* was the marker for low ARS score, and *REG4* is the marker for high ARS score. We further analyzed the differences in cellular features such as aging (Fig. S3F), EMT (Fig. S3G), cell migration (Fig. S3H), cell proliferation (Fig. S3I), *PI3K/AKT*/mTOR (Fig. S3J), *PI3K/AKT* activation (Fig. S3K), and *PI3K/AKT* signaling (Fig. S3L) between high and low ARS risk groups. The results indicate that cells in the high ARS risk group exhibit increased aging, proliferation, invasion, migration capabilities, and heightened *PI3K/AKT* signaling pathway activity.

### *EMP1* identified as the pivotal molecule in the ARS system

Upon further analysis of the differences in aging, migration, invasion, and G2M checkpoint characteristics between high- and low-ARS risk subgroups of PC cells, we discerned that cells from the high-risk primary PC, hepatic metastatic lesions, and adjacent normal tissues exhibited elevated aging, enhanced invasive-migratory capabilities, and higher G2M checkpoint scores (Fig. S4A). These findings underscore the potential of the ARS scoring system as a pivotal determinant in distinguishing aging and invasive-migratory capacities of PC cells. Among the genes constituting the ARS scoring system, *EMP1* manifested the highest baseline expression and was notably upregulated in PC patients aged 60 or older, suggesting a potential association between *EMP1* and aging (Fig. S4B). Furthermore, a significant correlation was observed between *EMP1* and the aging-related gene *KAT7* (*r* = 0.41, *p* < 0.001). These insights emphasize the significance of *EMP1* as a key molecule within the ARS scoring system.

### Differential ARS risk and *EMP1* levels in PC cells across trajectory stages

To elucidate the dynamic trends of single-cell ARS scores and *EMP1* expression during PC progression, cellular trajectory analysis was conducted (Fig. S5A, B). The analysis revealed that cells with varying ARS risks were situated at different stages of the PC developmental trajectory, indicating the dynamic alteration process of ARS scores in progressing PC cells. Further analysis revealed *EMP1* expression increased from initially low levels to higher of as PC cells progressed, with lineage 2 cells exhibiting a pronounced bimodal pattern (Fig. S5C). Additionally, cells were classified into six subgroups based on gene expression patterns, each characterized by uniquely expressed genes, suggesting potential roles of these genes during tumor progression. Moreover, as PC progressed, lineage 2 cells manifested heightened aging, augmented invasive-migratory capabilities, elevated G2M checkpoint scores, and enhanced *PI3K/AKT* pathway activity (Fig. S5D).

### Overexpression of *EMP1* in PC is associated with poor prognosis

**Figure 3:**
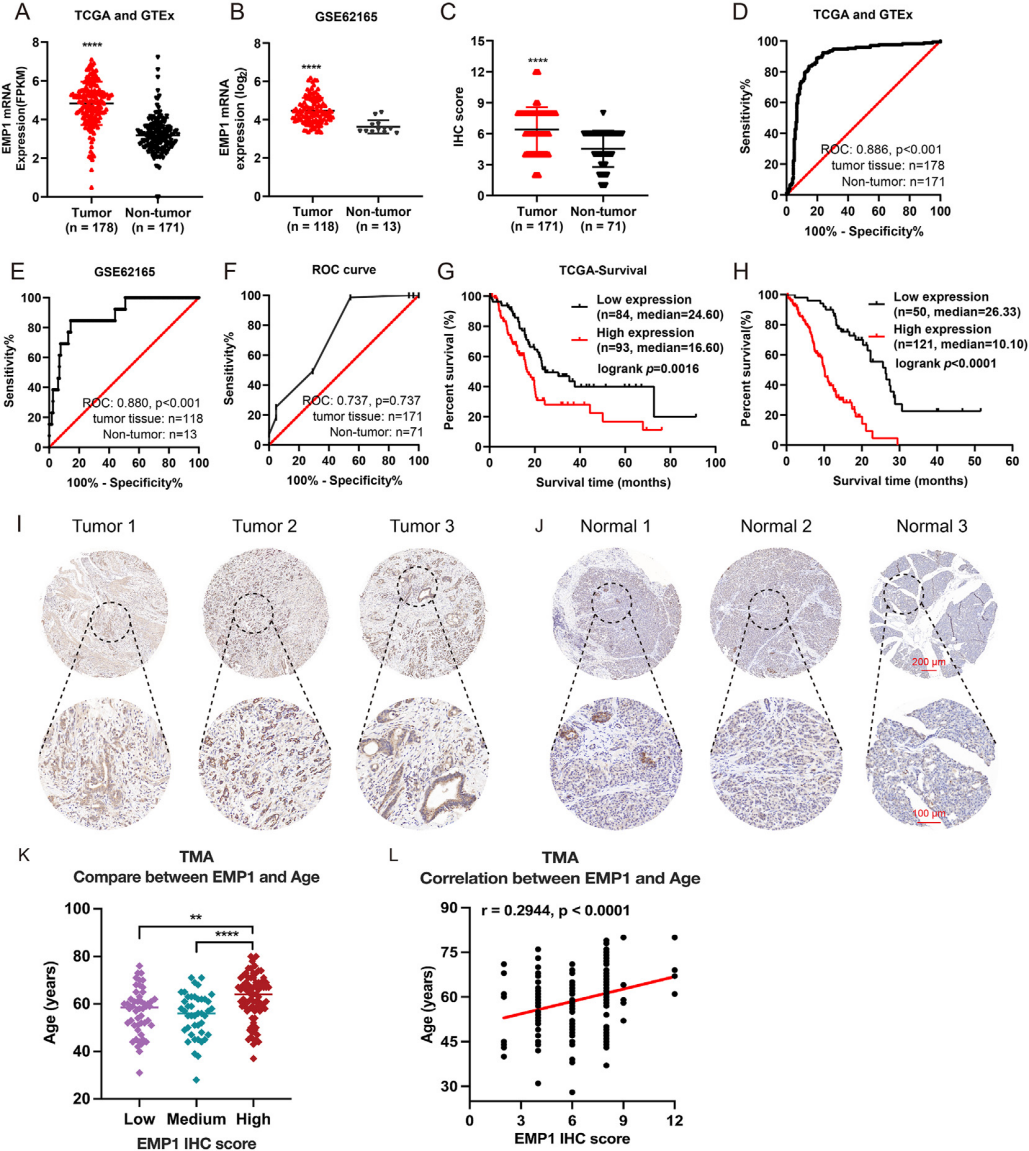
Elevated *EMP1* expression in pancreatic cancer (PC) correlates with poor patient outcomes. **(A**–**F)** Public databases (The Cancer Genome Atlas (TCGA) and genotype-tissue expression, GSE62165) and our tissue microarrays (TMA) cohorts provided the mRNA and protein expression profiles and associated receiver operating characteristic curves for *EMP1* in PC patients. **(G**–**H)** Kaplan-Meier plots demonstrated a negative correlation between the levels of *EMP1* expression and the overall survival duration of patients, as evidenced by datasets TCGA and our TMA cohorts. **(I, J)** Through immunohistochemical analysis, overexpression of EMP1 was observed in PC tissues (I) when compared to benign pancreatic samples (J). **(K)** By Subdividing the patients into three groups: high (*n* = 78), medium (*n* = 43), and low (*n* = 50) EMP1 expression, the dot plot showed the age difference between EMP1 high expression group and EMP1 medium expression group, the age difference between EMP1 high expression group and EMP1 low expression group, and age difference among three groups. **(L)** Spearman’s correlation analysis of EMP1 expression and age. ∗*p* < 0.05; ∗∗*p* < 0.01; ∗∗∗*p* < 0.001; ∗∗∗∗*p* < 0.0001.

Drawing from the TCGA, genotype-tissue expression, and GSE16515 public datasets, coupled with a TMA cohort comprising 171 PC tissues and 71 normal pancreatic tissues, we analyzed the mRNA and protein expression profiles of EMP1, along with the corresponding ROC curves (Fig. 3A–F, I–J). The data revealed a pronounced overexpression of EMP1 in PC tissues compared to its expression levels in non-tumorous tissues. Survival analyses using the Kaplan–Meier method on both the TCGA and TMA cohorts indicated that patients with high EMP1 expression exhibited poorer prognostic outcomes (Fig. 3G, H). Clinical feature analysis of the TMA cohort showed that the high EMP1 expression group had a more advanced stage of differentiation (Table S3). We further subdivided the patients into three groups based on EMP1 expression: high (*n* = 78), medium (*n* = 43), and low (*n* = 50), which was a more granular classification that allowed us to perform a more detailed investigation of EMP1 expression across different age groups. The results demonstrated a significant age difference between the EMP1 high expression group and the EMP1 medium expression group (*p* < 0.01), between the EMP1 high expression group and the EMP1 low expression group (*p* < 0.0001), and among the three groups (*p* < 0.0001) (Fig. 3K). Moreover, Spearman’s correlation analysis revealed a statistically significant positive relationship between EMP1 expression and age (*p* < 0.0001, *r* = 0.2944) (Fig. 3L). We also examined the relationship between EMP1 expression and prognosis by univariate and multivariate Cox proportional hazards analyses (Table S4). Variables with *p*-value <0.05 in the univariate Cox analysis were then incorporated into the multivariate regression model. The findings identified *EMP1* as an independent prognostic risk factor (HR = 4.401, *p* < 0.001). Collectively, EMP1’s elevated expression in PC tissues and these observations underscore its potential as an independent diagnostic and prognostic marker.

### *EMP1* enhances proliferation, migration, and invasion in PC cells *in vitro*

The pronounced upregulation of *EMP1* across multiple datasets, coupled with its association with adverse prognostic outcomes, strongly suggests that *EMP1* may function as a pivotal oncogene in PC. To decipher the potential biological role of *EMP1* in PC progression, we established cell models with either *EMP1* knockdown or overexpression in the AsPC-1 and PANC-1 cell lines.

**Figure 4:**
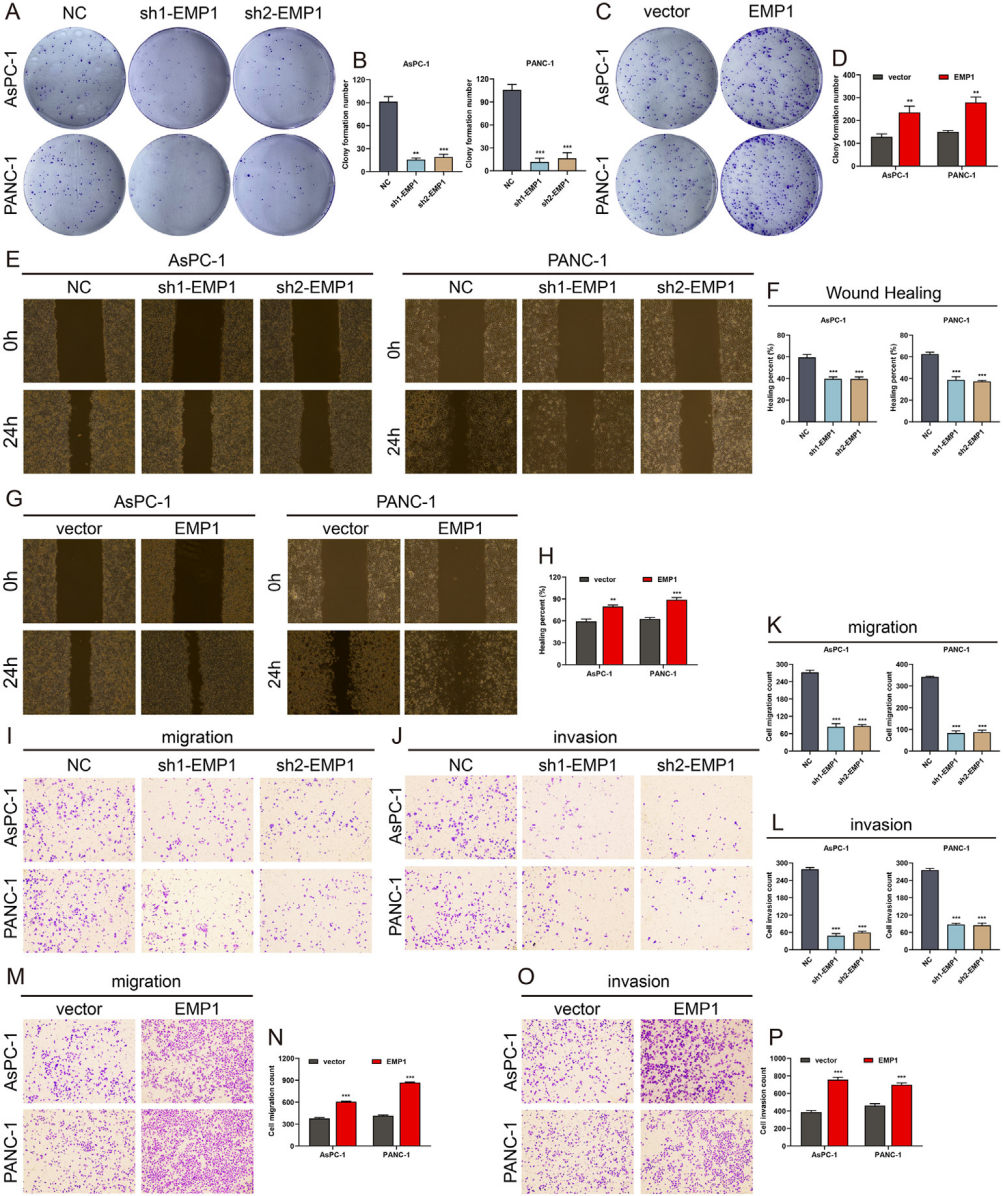
*EMP1* augments pancreatic cancer cell growth and enhances cell motility. **(A**–**D)** The proliferative capacity of *EMP1*-deficient (A, B) and *EMP1*-enriched cells (C, D) was assessed using a colony formation assay. **(E**–**H)** Scratch tests were employed to evaluate cell motility for the indicated cells. **(I**–**P)** Migration and invasion abilities of cells were ascertained through Transwell assays. Data are represented as mean ± SD from three independent experiments. ∗*p* < 0.05; ∗∗*p* < 0.01; ∗∗∗*p* < 0.001.

We conducted colony formation assays to evaluate the impact of *EMP1* modulation on the proliferative capacity of PC cells. The results revealed that PC cells with *EMP1* knockdown formed significantly fewer colonies, indicating a diminished cell proliferative capacity (Fig. 4A, B). Conversely, overexpression of *EMP1* led to an increased number of colonies, signifying enhanced cell proliferation capacity (Fig. 4C, D). We employed wound healing and transwell assays to assess the migratory and invasive capabilities of the treated cells. Overexpression of *EMP1* promoted cell migration, while its downregulation exhibited the opposite effect (Fig. 4E–H). Transwell invasion assays confirmed that overexpression of *EMP1* bolstered the invasive capacity of tumor cells, whereas its downregulation attenuated this ability (Fig. 4I–P). Collectively, these findings underscore that *EMP1* accelerates the proliferation, migration, and invasion of PC cells.

### *EMP1* augments the *PI3K/AKT* signaling cascade in PC *in vitro*

Based on the expression levels of *EMP1*, we stratified the TCGA-PAAD dataset into high- and low-expression groups and undertaken KEGG and GO enrichment analyses for differential genes. Our findings indicated a positive correlation between *EMP1* expression and the *PI3K/AKT* pathway. Moreover, *EMP1* was associated with cell metastasis-related pathways such as focal adhesion, ECM-receptor interaction, extracellular structure organization, and extracellular matrix organization (Fig. S6A, B). GSEA enrichment corroborated the positive association of *EMP1* expression with both the *PI3K/AKT* pathway and EMT (Fig. S6C, D). Spearman correlation analysis within the TCGA-PAAD dataset revealed that *EMP1* expression positively correlated with EMT marker scores and the *PI3K/AKT* signaling cascade (Fig. S6E, F).

Further exploration of the TCGA-PAAD database for correlations between *EMP1* gene expression and key functional phenotypic markers revealed significant positive associations of *EMP1* mRNA levels with proliferation markers such as *MMUT, PCNA*, and *MKI67*, as well as EMT-related markers like *N-cadherin, VIM, FN1, MMP2,* and *MMP9* (Fig. S6G–N). Figure S7 also highlighted a consistent expression trend between *EMP1* and EMT-related markers, including *CCNNB1, ACTA2, SNAI1, SNAI2, TWIST1, TWIST2, ZEB1,* and *ZEB2*.

**Figure 5:**
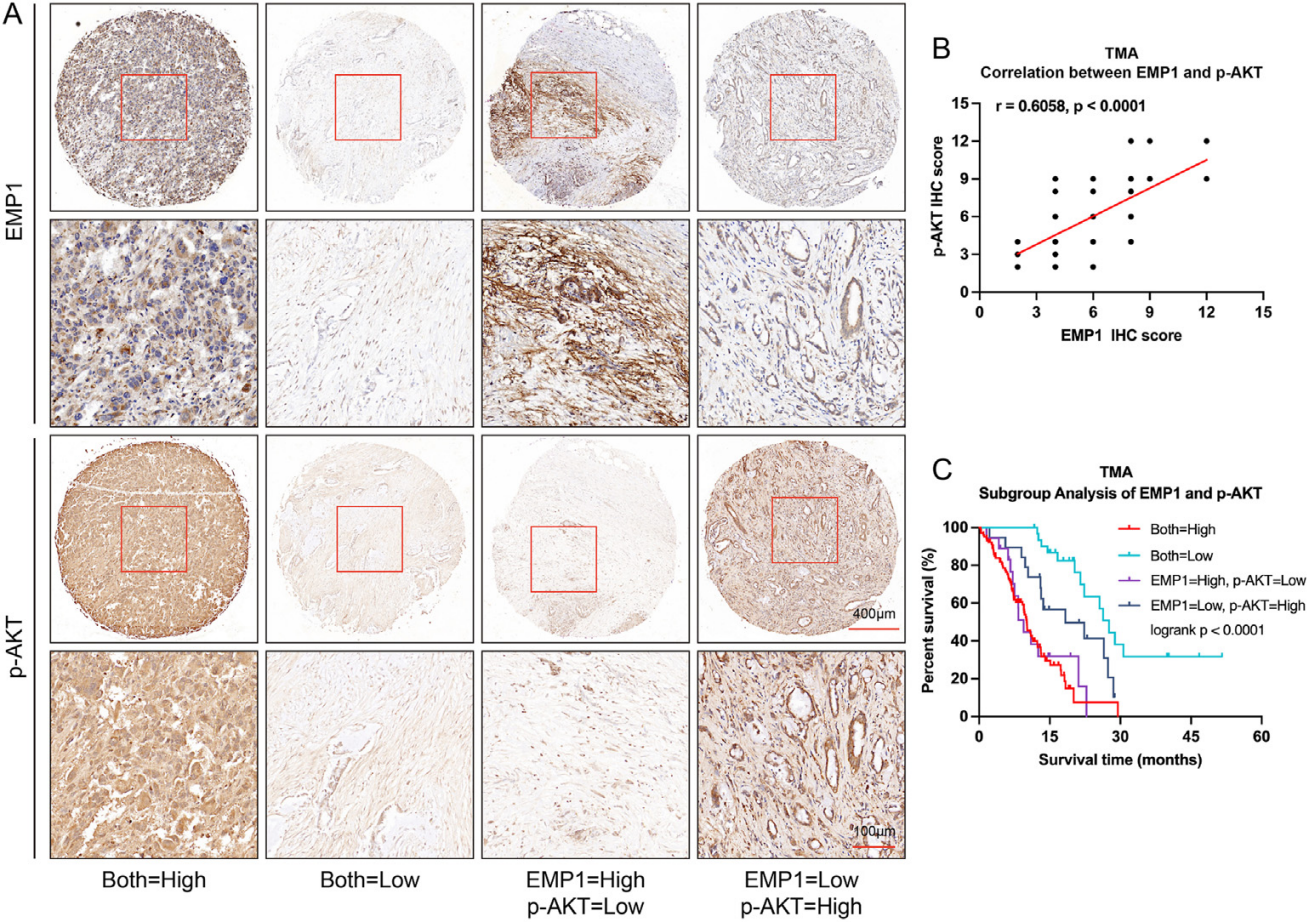
EMP1 protein expression shows a positive correlation with the PI3K/AKT signaling pathway in pancreatic cancer (PC) cells. **(A)** Representative images showcasing differential immunohistochemical analysis staining intensities for EMP1 and p-AKT in tissue samples (high denotes elevated expression; low indicates diminished expression). **(B)** Spearman correlation analysis elucidates the interrelation between EMP1 and p-AKT. **(C)** Kaplan–Meier survival assessment was performed on quartiles of PC patients, categorized by EMP1 and p-AKT expression levels, utilizing tissue microarrays cohorts (*n* = 171).

Protein-level analysis of the relationship between EMP1 and PI3K/AKT was conducted in a tissue TMA cohort derived from the same patient group. Spearman correlation analysis demonstrated a significant positive association between the expression levels of EMP1 and p-AKT in PC tissues (Fig. 5A, B). Furthermore, subgroup Kaplan–Meier survival analysis indicated that patients with concurrent low expression of EMP1 and p-AKT exhibited the most favorable prognosis (Fig. 5C). In summary, our findings underscore a positive correlation between EMP1 and PI3K/AKT expression, suggesting that EMP1 may modulate the clinical outcomes of PC patients through its interaction with the PI3K/AKT pathway.

### *EMP1* correlated with malignant behaviors at the single-cell level

**Figure 6:**
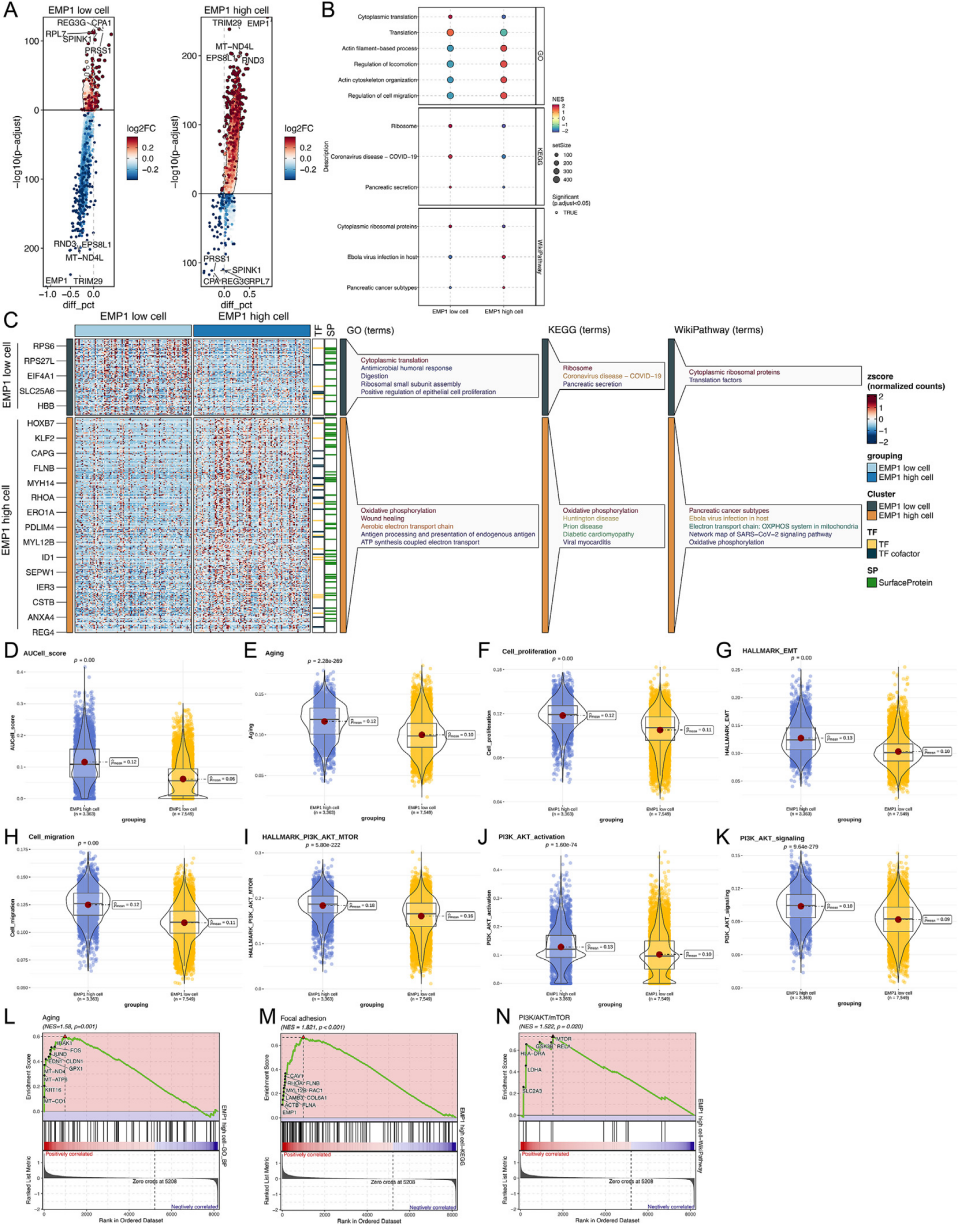
Different characteristics of high and low *EMP1* expression of pancreatic cancer cells at the single-cell level. **(A)** Differentially expressed genes of high and low *EMP1* expression cells. **(B)** Pathway enrichment of high and low *EMP1* expression cells. **(C)** Gene expression characteristics of high and low *EMP1* expression cells. **(D**–**K)** Violin plot of age-related score AUCell score (D), aging (E), cell proliferation (F), EMT (G), cell migration (H), *PI3K/AKT*/*mTOR* (I), *PI3K/AKT* activation (J), and *PI3K/AKT* signaling (K) degree between high and low *EMP1* expression cells. **(L**–**N)** Gene Set Enrichment Analysis of aging, focal adhesion, and *PI3K/AKT*/*mTOR* pathway between high and low *EMP1* expression cells.

Subsequently, we sought to elucidate the differential malignant behaviors of PC cells with varying *EMP1* expression at the single-cell level, aiming to further discern the potential mechanisms underpinning *EMP1*’s role. Single-cell sequencing data revealed *EMP1*-overexpressing tumor cells exhibited a distinct gene expression profile, notably upregulating genes such as *TRIM29* and *RND3* (Fig. 6A), and were significantly enriched in pathways related to cell migration and cytoskeletal dynamics (Fig. 6B), suggesting *EMP1*’s association with tumor cell motility. Comparative analysis between high and low *EMP1*-expressing tumor cells revealed overexpression of genes encoding transcription factors and surface proteins in the former, predominantly enriched in oxidative phosphorylation pathways (Fig. 6C). Moreover, tumor cells with elevated *EMP1* demonstrated heightened ARS risk, aging propensity, invasive and migratory capabilities, proliferative capacity, and *PI3K/AKT* pathway activity (Fig. 6D–K). Further GSEA analysis indicated that high *EMP1*-expressing tumor cells were significantly enriched in aging (NES = 1.58, *p* = 0.001), focal adhesion (NES = 1.821, *p* < 0.001), and *PI3K/AKT/mTOR* pathways (NES = 1.522, *p* = 0.001) (Fig. 6L–N). These findings suggest that *EMP1* potentially augments malignant processes such as aging, proliferation, invasion, and migration in PC cells, possibly mediated through the modulation of the *PI3K/AKT* pathway.

### *EMP1* modulates malignant behaviors in PC via the *PI3K/AKT* signaling pathway *in vitro*

**Figure 7:**
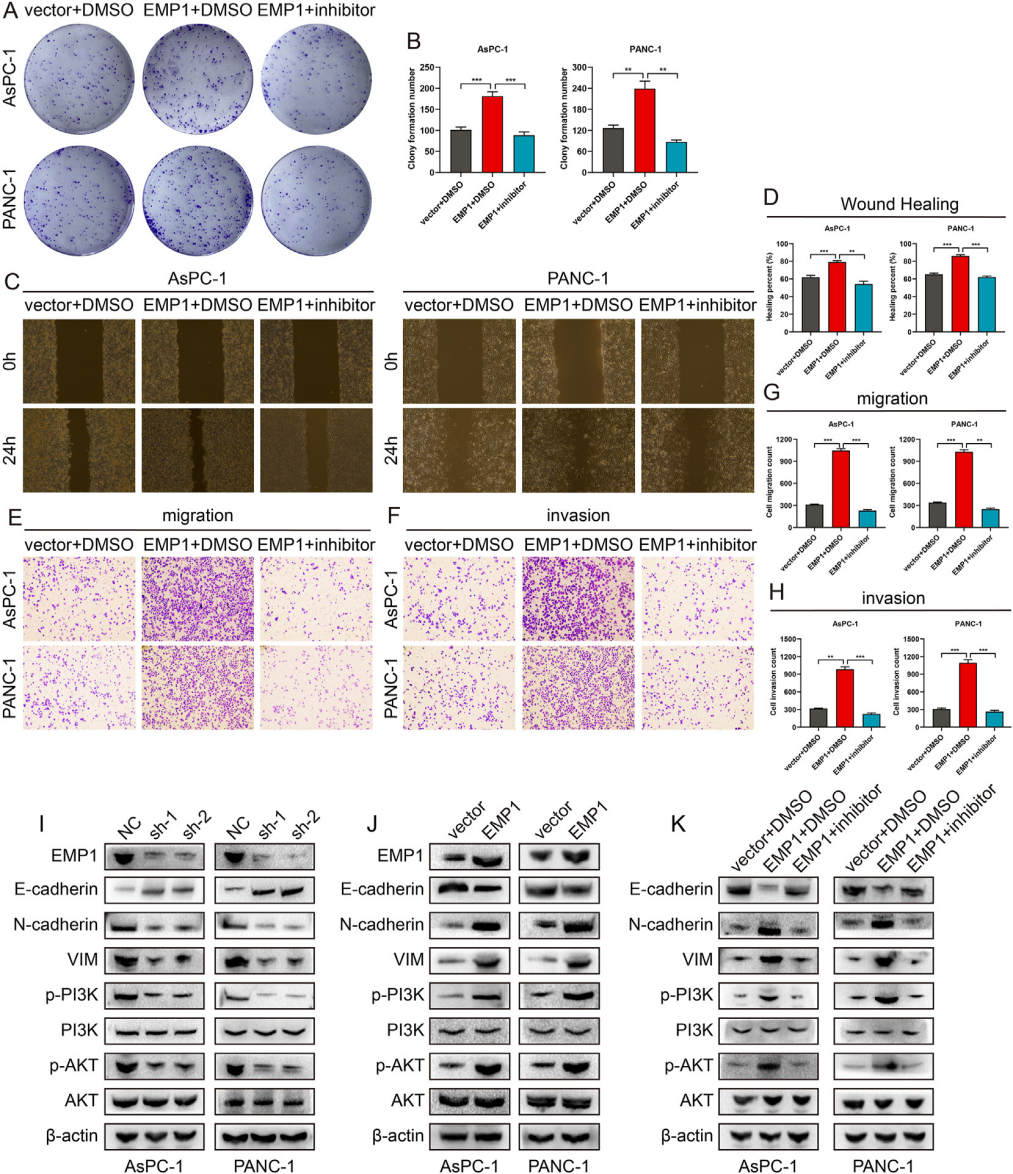
*EMP1* influences tumor growth, migration, and invasive characteristics through the *PI3K/AKT* signaling cascade in pancreatic cancer cells. **(A**, **B)** Following EMP1 augmentation or *PI3K/AKT* pathway inhibitor application, the colony-forming potential was assessed using a colony-formation assay. **(C**, **D)** Scratch assays were employed to assess the movement dynamics of the tested cells. **(E**–**H)** Migration and invasion capacities of the cells were ascertained using Transwell assays. Data are depicted as mean ± SD, derived from three separate experiments. **(I**–**K)** Western blotting was conducted to determine the expression levels of EMP1, E-cadherin, N-cadherin, VIM, p-PI3K, total PI3K, p-AKT, and total AKT in the designated cells. β-actin was utilized as a normalization control. ∗*p* < 0.05; ∗∗*p* < 0.01; ∗∗∗*p* < 0.001.

To elucidate the regulation between *EMP1* and the *PI3K/AKT* pathway, cells overexpressing *EMP1* were treated with the *PI3K/AKT* pathway inhibitor, LY294002. This intervention was designed to assess the influence of *PI3K/AKT* on *EMP1*-induced cellular proliferation, migration, and invasion. As depicted in Figure 7A and B, LY294002 effectively mitigated the proliferative effects induced by *EMP1*. Scratch assays and transwell rescue experiments indicated that inhibiting *PI3K/AKT* counteracted the *EMP1*-mediated migration and invasion of PC cells (Fig. 7C–H). Western blot analyses revealed that upon EMP1 knockdown, there was a downregulation of p-PI3K, p-AKT, N-cadherin, and VIM, and an upregulation of E-cadherin in PC cells (Fig. 7I). Conversely, overexpression of EMP1 activated p-PI3K, p-AKT, N-cadherin, and VIM, while suppressing E-cadherin expression (Fig. 7J). Inhibiting PI3K/AKT in PC cells with elevated EMP1 expression reversed the oncogenic effects of EMP1 (Fig. 7K). In summary, our findings underscore the regulatory role of EMP1 in promoting malignant behaviors in PC through the activation of the PI3K/AKT signaling cascade.

### *EMP1* potentiates PC’s oncogenicity through the *PI3K/AKT* pathway *In vivo*

**Figure 8:**
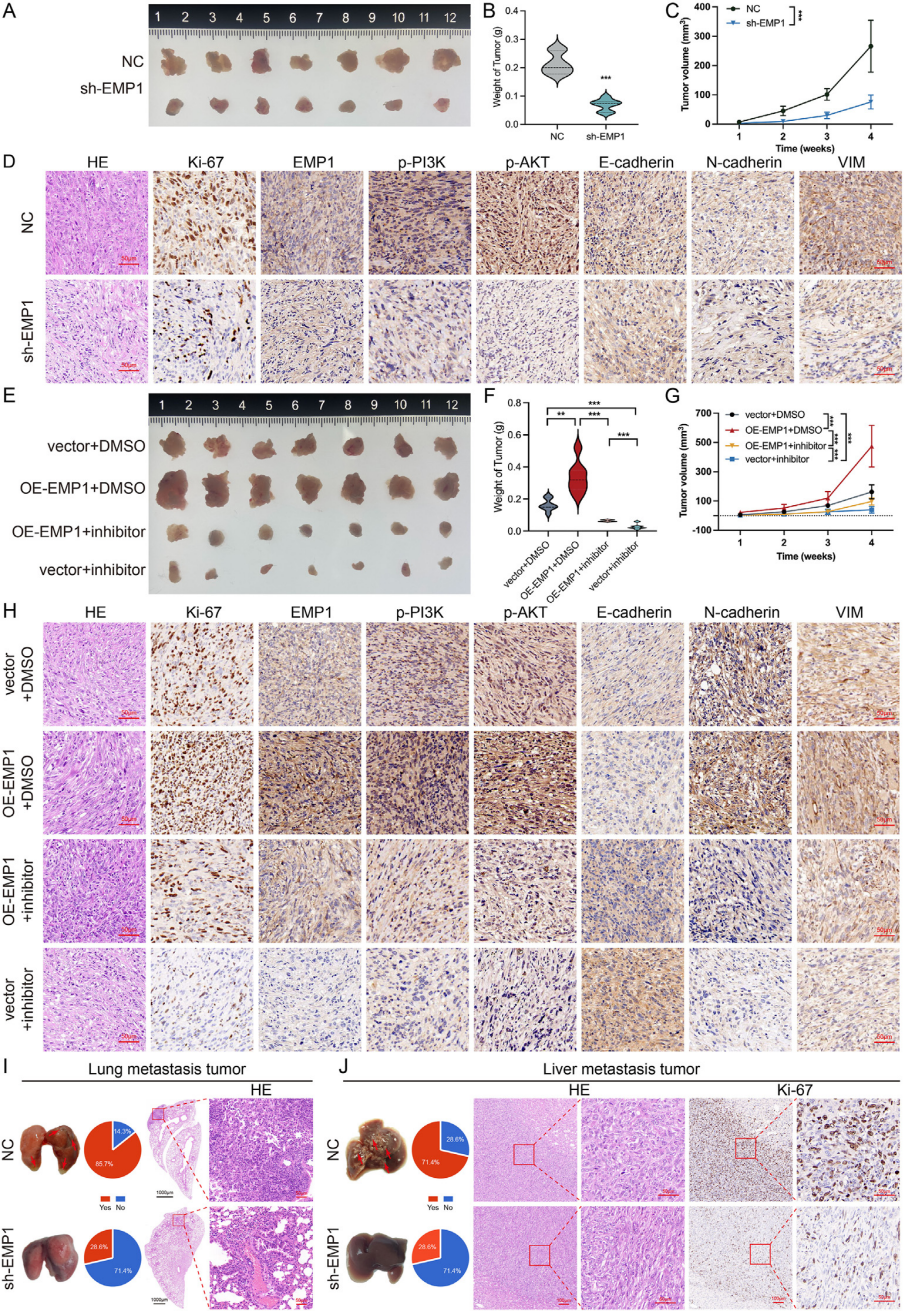
*EMP1* mediates pancreatic cancer (PC)’s oncogenic activities *in vivo* through the *PI3K/AKT* pathway. **(A)** Illustrative xenograft images from specified mice cohorts. **(B)** Mean tumor weight. **(C)** Tumor metrics were measured 4 weeks after cell introduction. **(D)** Hematoxylin-eosin (HE) and immunohistochemical analysis (IHC) analyses of tumor samples. **(E)** Tumor weight delineating the influence of EMP1 amplification and PI3K/AKT pathway inhibition (50 mg/kg, intraperitoneal injection, every 3 days) on selected cohorts. **(F)** Average tumor mass. **(G)** Periodic tumor dimension evaluations. **(H)** HE and IHC staining of tumor slices. **(I)** Targeting *EMP1* diminishes pulmonary metastasis of PC cells *in vivo*. This section presents images of pulmonary micrometastatic sites in both the control (NC) and *EMP1*-suppressed groups (sh-*EMP1*), a comparative analysis of pulmonary metastasis ratios among the groups (*n* = 7), and HE-stained images from a pulmonary metastasis model developed through tail vein inoculation of PC cells. **(J)***EMP1* targeting reduces hepatic metastasis of PC cells *in vivo*. This section includes images of hepatic micrometastatic sites in both the NC and sh-*EMP1* groups, a comparative analysis of hepatic metastasis ratios across the groups (*n* = 7), and HE and Ki-67 stained visuals of hepatic micrometastatic sites originating from *in situ* PC model. HE: Hematoxylin-eosin; OE: overexpression. ∗∗*p* < 0.01; ∗∗∗*p* < 0.001.

To elucidate the physiological effects of *EMP1 in vivo*, we employed lentiviral vectors for both knockdown and overexpression of *EMP1* in Panc02 cells, subsequently establishing subcutaneous, pulmonary metastasis, and orthotopic pancreatic liver metastasis models in C57BL/6J mice. During the *in vivo* assessments, *EMP1* attenuation manifested a pronounced decrement in the metrics of subcutaneous tumor xenografts, both in terms of volume and mass (Fig. 8A–C). In stark contrast, the amplification of *EMP1* expression precipitated a substantial surge in the aforementioned tumor parameters relative to baseline controls (Fig. 8E–G). The tumorigenic propensity conferred by heightened *EMP1* was effectively curtailed by the *PI3K/AKT* pathway antagonist (Fig. 8E–G). In the *EMP1*-suppressed group, a reduction in pulmonary metastasis from the tail vein model and hepatic metastasis from orthotopic pancreatic cancer was observed compared to the control group (Fig. 8I, J). Histological analysis of the xenograft specimens highlighted a direct correlation between EMP1 levels and markers such as p-PI3K, p-AKT, Ki-67, N-cadherin, and VIM, while an inverse association was noted with E-cadherin (Fig. 8D, H, J; Fig. S8 A–C).

In essence, our research suggested that *EMP1* amplifies the tumorigenic attributes of PC both *in vitro* and *in vivo*, encompassing facets like cellular proliferation and metastatic potential orchestrated through the *PI3K/AKT* molecular conduit (Fig. S9).

## Discussion

PC is a highly aggressive gastrointestinal malignancy with a dismal prognosis, characterized by an extremely low 5-year survival rate.^31^, ^32^, ^33^ Compared to patients with late-stage PC, curative surgical resection is the main treatment for resectable PC patients. At the same time, there are essential differences in prognosis between resectable and unresectable PC patients. Therefore, predicting prognosis in resectable PC patients is an important research topic. Early intervention for patients with a potentially poor prognosis after surgery can effectively prolong the survival time of resectable PC patients. In this study, we identified a prognostic prediction model suitable for resectable PC patients and identified key age-related drivers of *EMP1*. In actual application, transcriptome sequencing or ARS gene expression panel detection can be performed on surgically resected PC tissues to predict postoperative patient survival and enable early intervention and timely adjustment of treatment regimens to ultimately prolong patient postoperative survival. In addition, *EMP1* was also identified as an independent risk factor for poor prognosis in pancreatic cancer patients after surgery. In the future, targeted therapeutic drugs against this molecule can be developed to improve the prognosis of resectable PC patients.

The process of organismal aging plays a pivotal role in tumorigenesis and progression. Recent studies have underscored the association between PC development, tumor microenvironment formation, and patient age.^34^ Advancing age precipitates systemic and microenvironmental changes that appear to facilitate tumor growth and metastasis through several mechanisms, ultimately enabling more aggressive cancer phenotypes. Elucidating the precise contributions of host aging dynamics to PC evolution has important implications for prognostication and the development of age-specific anti-cancer interventions. Thus, we developed an ARS system using bulk RNA-sequence data from PC tissues and innovatively applied this model at the single-cell level. This model accurately predicts cellular aging status and associates aging vitality with tumor cell proliferation, migration, and EMT processes.

There exists a profound link between patient organismal aging and tumor metastasis. Age-related alterations in the tumor microenvironment potentially facilitate metastatic progression, encompassing changes in secretory factors, biophysical alterations, and shifts in matrix and immune cell populations.^35^ Interactions between senescent cell types in the aging tumor microenvironment and tumor cells can influence tumor metastasis and therapeutic drug responses.^36^ These findings suggest that the organismal aging process, which is accompanied by extensive gene expression alterations, potentially influences the overall cellular gene expression pattern and thereby affects tumor metastasis initiators or promoters. Our study reveals that, the ARS system, an aging prediction model, not only forecasts the prognosis of PC patients at the tissue level, but also differentiates the overall EMT activity of tumor tissues. Further, at the single-cell level, the ARS system can effectively predict cellular aging status and tumor cell EMT vitality, showcasing promising applicability. Our results also corroborate that, at both the tissue and single-cell levels, as organismal aging and cellular senescence ensue, the EMT capability of PC intensifies, predisposing to metastasis.

PC progression has been intricately linked to malignant biological behaviors, including cell proliferation, migration, and invasion. Previous studies have identified various molecules that promote tumor progression by modulating these malignant behaviors in PC cells. For instance, the *TRIM15-APOA1-DLR* axis potentially inhibits PC metastasis by blocking triglyceride synthesis.^37^ *CD74* enhances the neuroinvasive potential of PC cells and mediates neural plasticity via the *AKT/EGR-1/GDNF* axis.^38^ *IFIT1* promotes cell proliferation, migration, and invasion in PC cells by activating the Wnt/β-catenin pathway.^39^ In our study, we identified *EMP1* as a key factor in the aging process of PC and as an integral component of the ARS system. Both *in vivo* and *in vitro* experiments demonstrated a strong association between *EMP1* and malignant biological behaviors in PC. Furthermore, tissue microarray-based clinical correlation studies revealed that EMP1 expression levels significantly correlate with OS in PC patients, serving as an independent prognostic factor. These findings suggest that *EMP1* acts as an oncogene in PC, and targeting this molecule could have potential therapeutic implications for inhibiting tumor growth and reversing metastasis.

EMP1, an epithelial membrane protein, exhibits oncogenic properties across various malignancies. In ovarian cancer, overexpressed *EMP1* promotes tumor cell proliferation, invasion, and EMT via the MAPK signaling pathway.^25^ The absence of *EMP1* stimulates bladder cancer cell migration by activating the PPARγ signaling pathway.^40^ Elevated *EMP1* expression in osteosarcoma tissues is closely associated with lymphatic and distant metastasis, stimulating malignant progression via the *IRX2/MMP9* axis.^22^ Our subsequent investigations revealed that *EMP1* exerts its oncogenic effects in PC through the *PI3K/AKT* pathway *in vitro* and *in vivo*. Although prior research has underscored *EMP1*’s involvement in glioma^26^ and glioblastoma^20^ via the *PI3K/AKT* mechanism, our study uniquely identifies *EMP1* as an age-related factor that augments the oncogenic activities of PC via the *PI3K/AKT* pathway, both *in vitro* and *in vivo*.

The *PI3K/AKT* signaling pathway is an intracellular transduction mechanism that responds to extracellular cues, promoting metabolism, proliferation, cell survival, growth, and angiogenesis, and is intimately associated with the malignant behaviors of tumors.^41^ *FAM126A* interacts with *ENO1* and mediates PC proliferation and metastasis via the *PI3K/AKT* pathway.^42^ Activation of the glucagon-like peptide-1 receptor inhibits tumorigenesis and metastasis of PC cells through the *PI3K/AKT* pathway.^43^ Furthermore, *PI3K/AKT* has been intricately linked to PC cellular senescence.^44^ Both *in vivo* and *in vitro* results from our study indicate that the oncogenic effects of *EMP1* in PC can be reversed by *PI3K/AKT* pathway inhibitors, suggesting the potential for combinatorial therapeutic strategies targeting *EMP1* and *PI3K/AKT* to treat PC, inhibit tumor metastasis, delay disease progression, and potentially improve patient prognosis.

Our study, using both *in vivo* and *in vitro* validations, elucidated that the aging-associated factor *EMP1* in PC modulates tumor proliferation, invasion, and metastasis via the *PI3K/AKT* signaling pathway. A limitation of our study is the lack of detailed exploration into the precise mechanisms and processes by which *EMP1* impacts the *PI3K/AKT* pathway. Besides, the age-related changes relevant to cancer prognosis occur in the host microenvironment and supporting cells rather than the cancer cells themselves. The senescence of immune cells and the age-related deterioration of host systems probably have more impact. In order to more accurately determine the most critical aging-associated molecules in the tumor cells themselves, we deliberately removed components of the tumor microenvironment, such as immune cells and stromal cells, during single-cell analysis and only retained the tumor cells for subsequent analysis. However, this does not mean that immune cells, stromal cells, and other components in the tumor microenvironment of PC do not change during aging. Future research will focus on elucidating the specific modulatory effects of *EMP1* on the *PI3K/AKT* pathway and its role in remolding the PC immune microenvironment.

This study established an aging-based prognostic model for PC and identified *EMP1* as an oncogenic factor that promotes EMT in tumor cells during the aging process of resectable PC patients. We discover that *EMP1* regulates PC proliferation, invasion, and metastasis via the *PI3K/AKT* signaling pathway. The findings of this study hold promise for the development of targeted therapeutics addressing aging and metastatic processes in PC (Fig. S9).

## Funding

This work was sponsored by 10.13039/501100005230Natural Science Foundation of Chongqing, China (No. CSTB2022NSCQ-MSX1339 to J.F.Z.; CSTB2022NSCQ-MSX1273 to J.Y.G.) and Foundation of Key Laboratory of Tumor Immunology and Pathology (Army Medical University), Ministry of Education (No. 2023jsz910 to J.F.Z.); 10.13039/100014717National Natural Science Foundation of China (No. 82203165 to J.Y.G.; 82072723, 82103249 to H.Z.W.); Chongqing medical scientific research project (Joint project of Chongqing Health Commission and Science and Technology Bureau) (No. 2022MSXM031 to J.F.X.); and Chongqing Technology Innovation and Application Development Special Key Project (No. CSTB2022TIAD-KPX0170 to H.Z.W.).

## CRediT authorship contribution statement

**Junfeng Zhang:** Writing – original draft, Visualization, Funding acquisition, Formal analysis, Data curation, Conceptualization. **Jianyou Gu:** Writing – original draft, Resources, Investigation, Data curation, Conceptualization. **Tao Zhang:** Validation, Software, Data curation, Conceptualization. **Renpei Xia:** Methodology, Investigation. **Jianbo Li:** Methodology, Investigation. **Mingda Tan:** Methodology, Investigation. **Yongjun Yang:** Methodology, Investigation. **Jifeng Xiang:** Methodology, Investigation. **Bin Xie:** Methodology, Investigation. **Rong Tang:** Methodology, Investigation. **Wangge Li:** Methodology, Investigation. **Xianxing Wang:** Writing – review & editing, Supervision. **Shixiang Guo:** Writing – review & editing, Conceptualization. **Huaizhi Wang:** Writing – review & editing, Supervision, Funding acquisition, Conceptualization.

## Data availability

All data supporting the results of this study are available in the public databases mentioned in the article, as well as in additional files.

## Conflict of interests

The authors have no competing interests to declare.
