# Supplementary material for: The role of the aging process and related factor EMP1 in promoting progression of resectable pancreatic cancer
Source: Genes Dis. 2024 Dec 15;12(5):101490. doi: 10.1016/j.gendis.2024.101490 (PMC12221743; doi:10.1016/j.gendis.2024.101490)
Supplement: Multimedia component 1 [file mmc1.docx]

**Supplementary Figures**

**
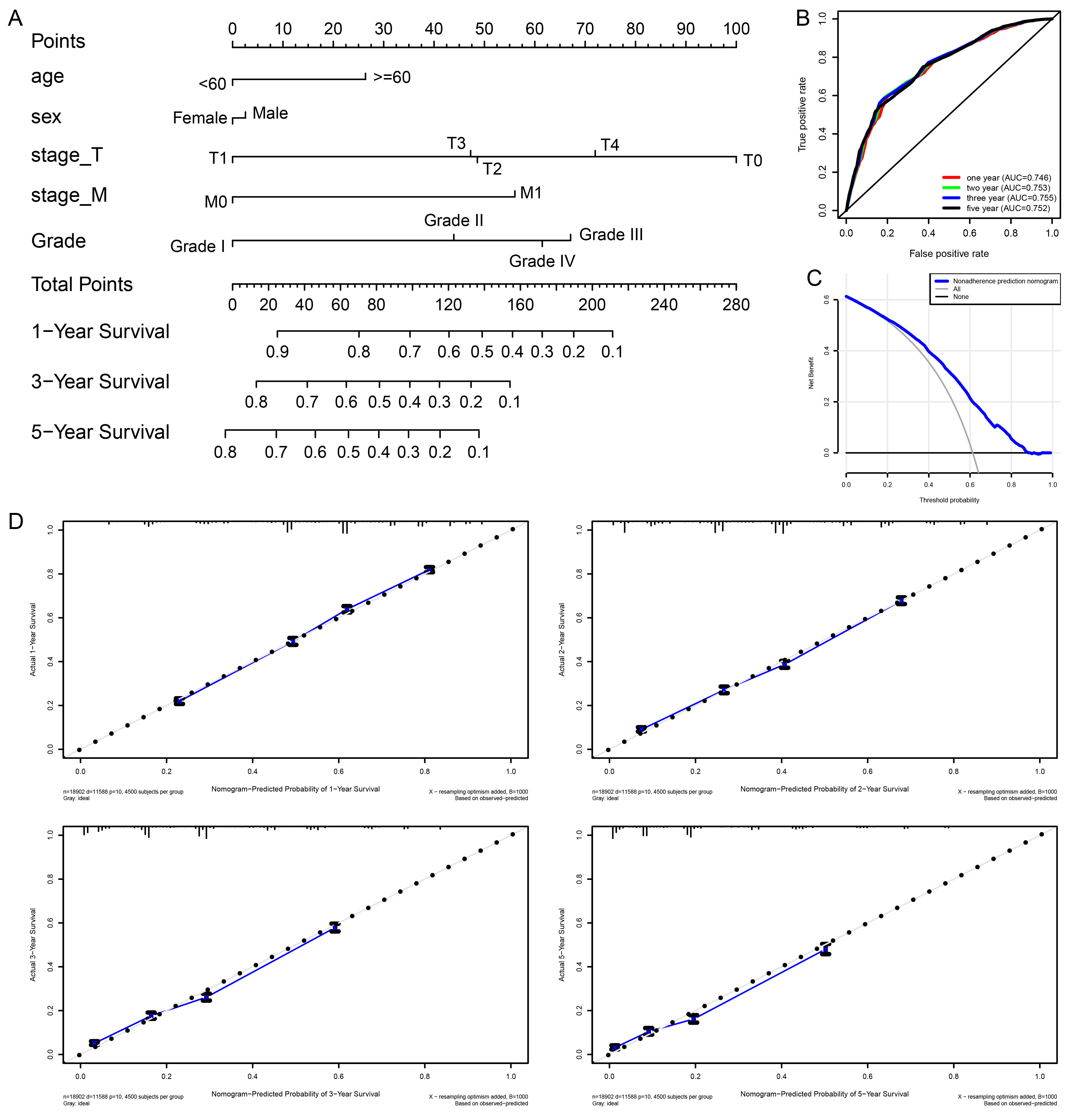
**

**Supplementary Figure 1. Development and verification of the SEER model for PC prognosis prediction.** (A) A prognostic nomogram for PC was formulated based on the extensive SEER dataset. (B) The temporal ROC curve illustrating the performance of the prediction model. (C) Decision curve analysis showcasing the efficacy of the nomogram. (D) Calibration plots at 1, 2, 3, and 5-year intervals demonstrate the nomogram's reliable calibration for PC within the SEER dataset.


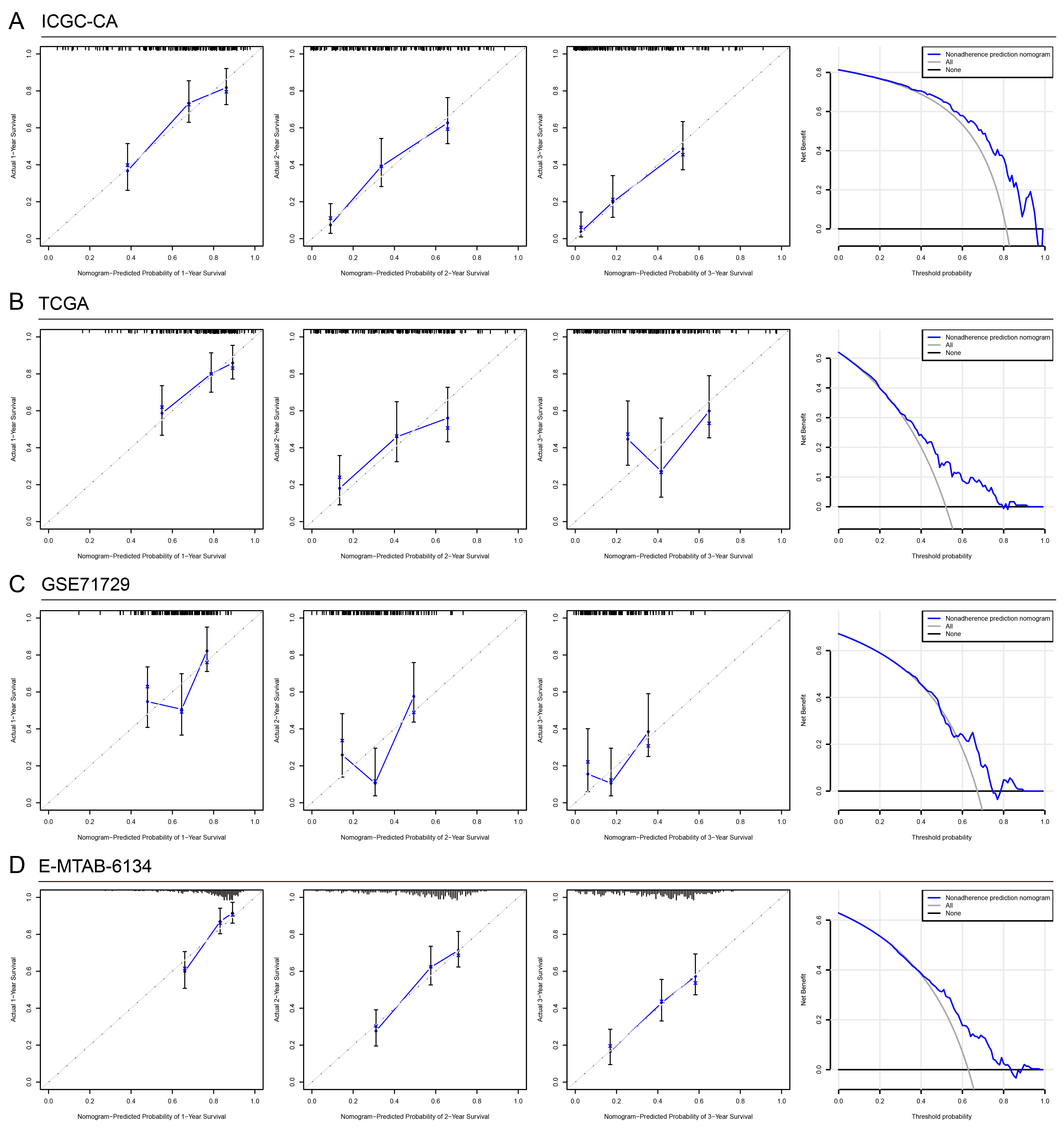


**Supplementary Figure 2. Identification and validation of ARS.** (A-D) Calibration curves for the nomogram illustrate its reliable calibration performance across the training and validation cohorts for 1, 2, and 3-year intervals. The decision curve for the nomogram is also depicted.


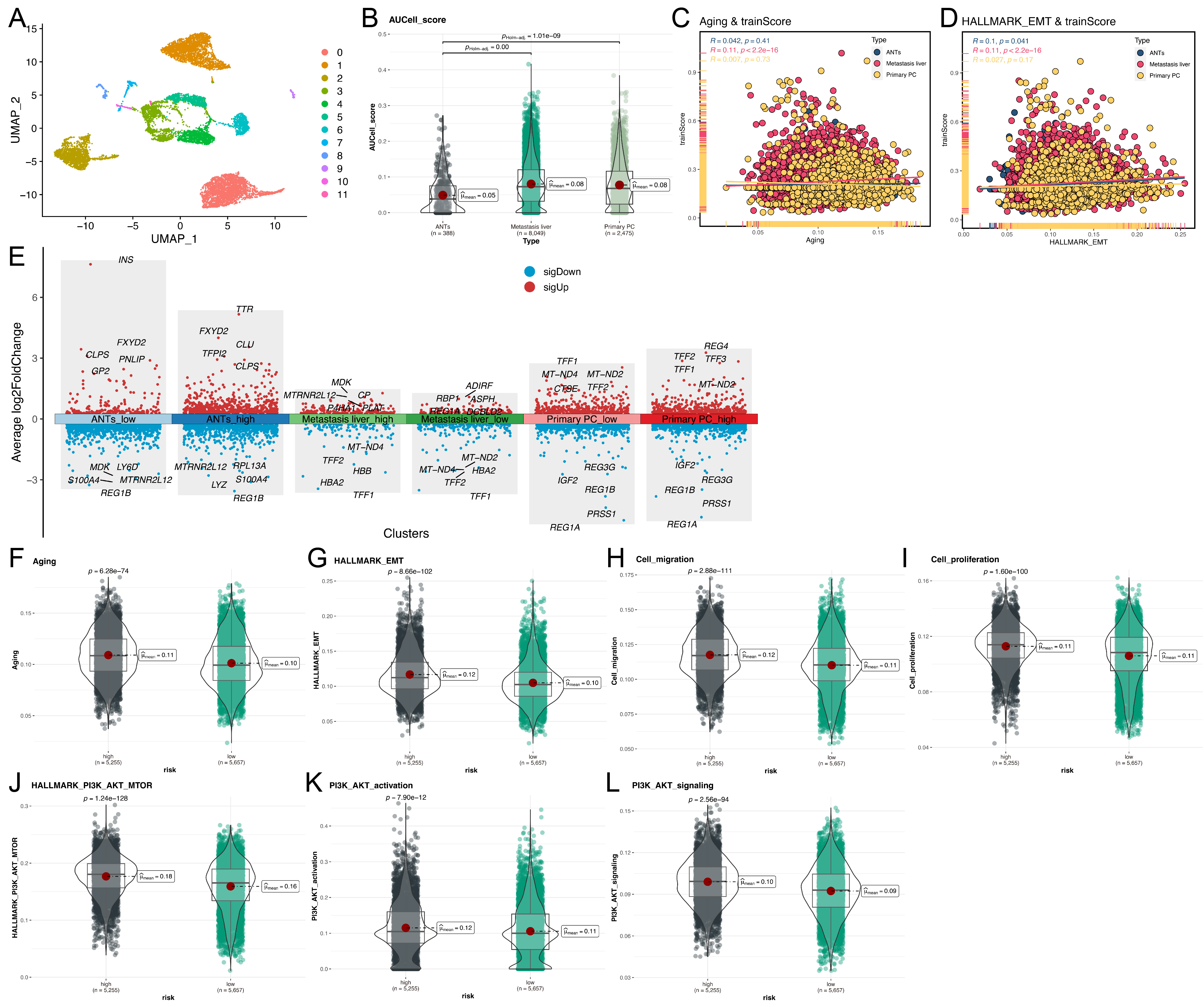


**Supplementary Figure 3. Application of ARS system in single-cell level.** (A) UAMP clustering of scRNA-seq data of primary PC, metastasis PC, and ANT tissue. (B) Violin plot of AUCell scoring of primary PC, metastasis PC, and ANT tissue using ARS genes. (C) Scatter plot of relation between ARS score and aging degree. Scatter plot of relation between ARS score and EMT degree. (D) Differential expressed genes of high and low ARS risk cells. (F-L) Violin plot of aging (F), EMT (G), cell migration (H), cell proliferation (I), *PI3K/AKT/mTOR* (J), *PI3K/AKT* activation (K), and *PI3K/AKT* signaling (L) degree between high and low ARS risk cells.


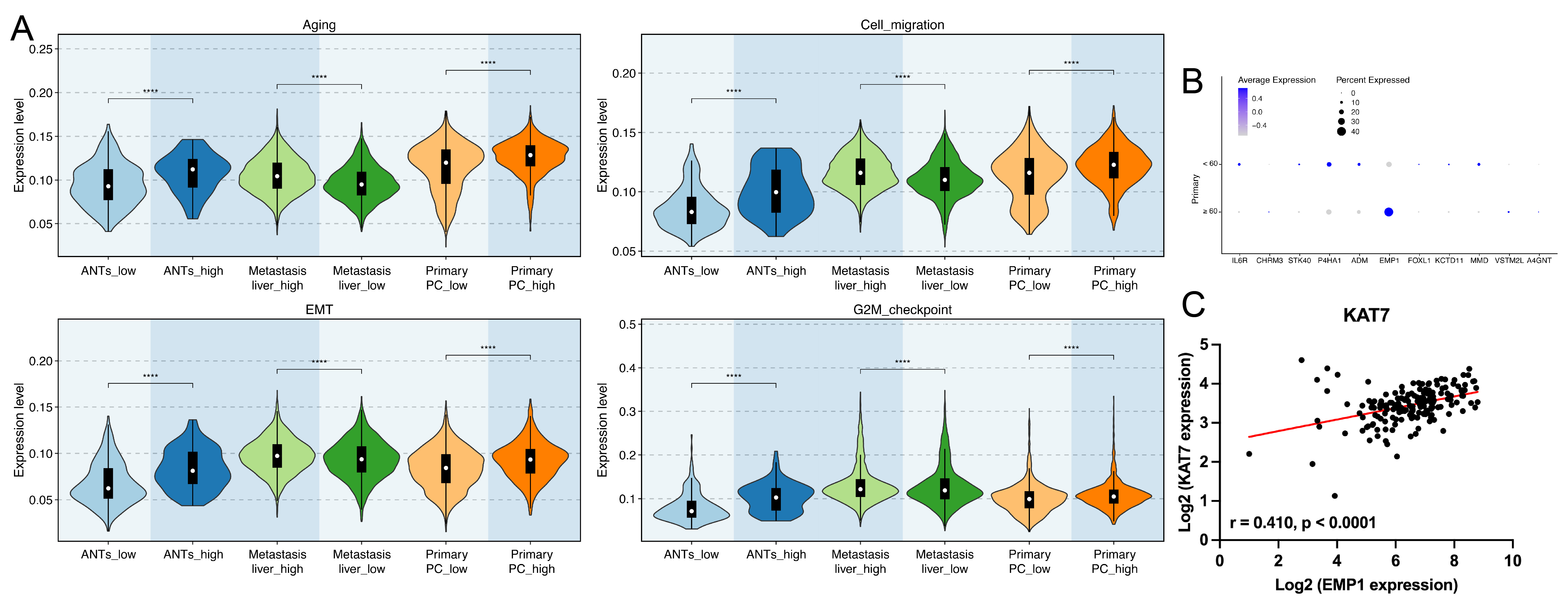


**Supplementary Figure 4. EMP1 may associate with malignant process of PC.** (A) Violin plot of aging, EMT, cell migration, and G2M checkpoint degree in high and low ARS risk cells in primary PC, metastasis PC, and ANT subgroups. (B) Dot plot of ARS related gene expression in older and younger PC patients. (C) The correlation between *KAT7* expression and *EMP1* expression in PC patients of TCGA dataset.


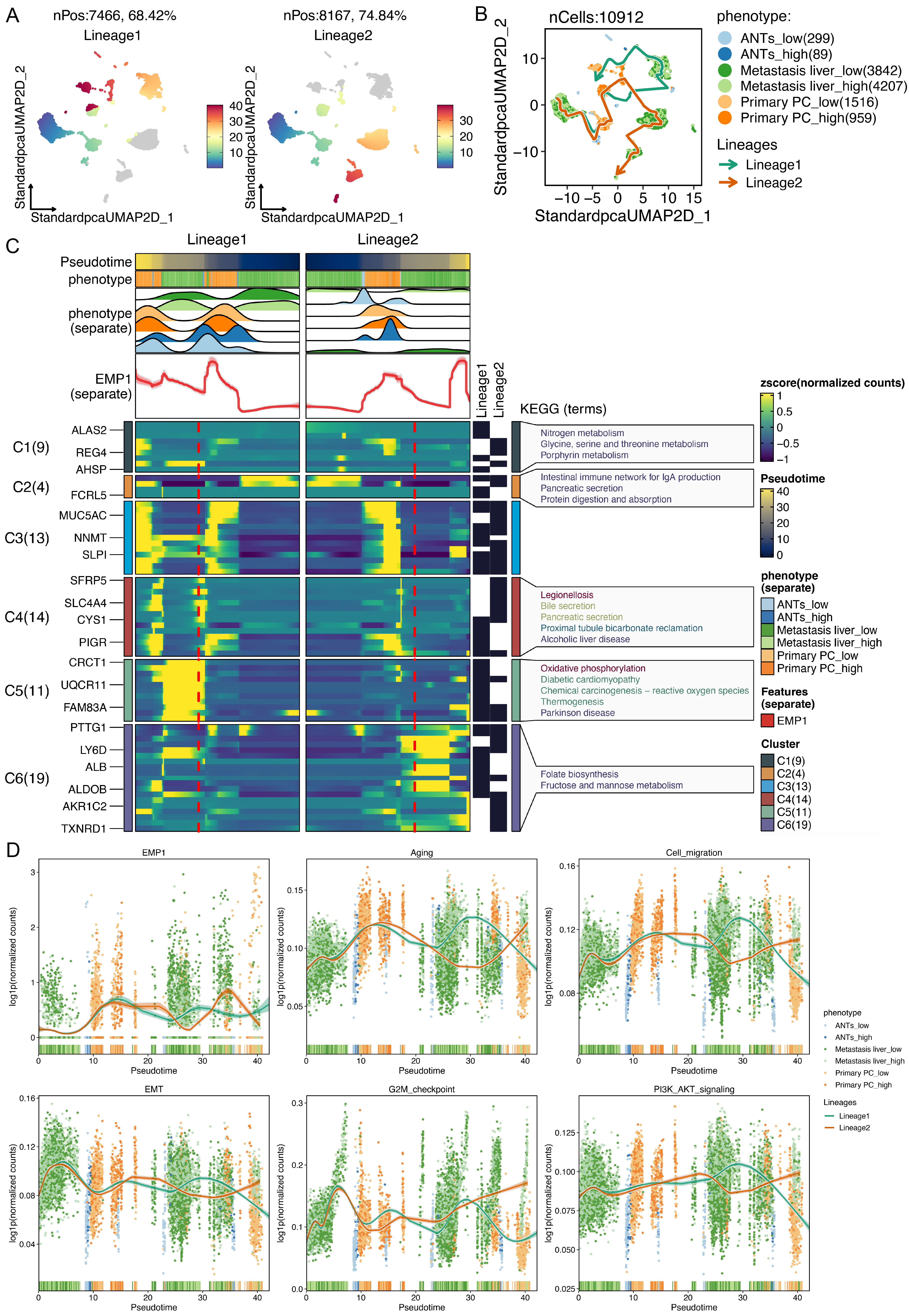


**Supplementary Figure 5. Specific trajectory in the evolution of PC.** (A) Trajectory analysis of primary PC, metastasis PC, and ANT cells. (B) Trajectory analysis of primary PC, metastasis PC, and ANT cells and visualization of high and low ARS risk subgroups. (C) Heatmap of characteristics in different lineages cells. (D) Trend plot of *EMP1* expression, aging, EMT, cell migration, G2M checkpoint, and *PI3K/AKT* signaling degree of different lineages cells during pseudotime going.


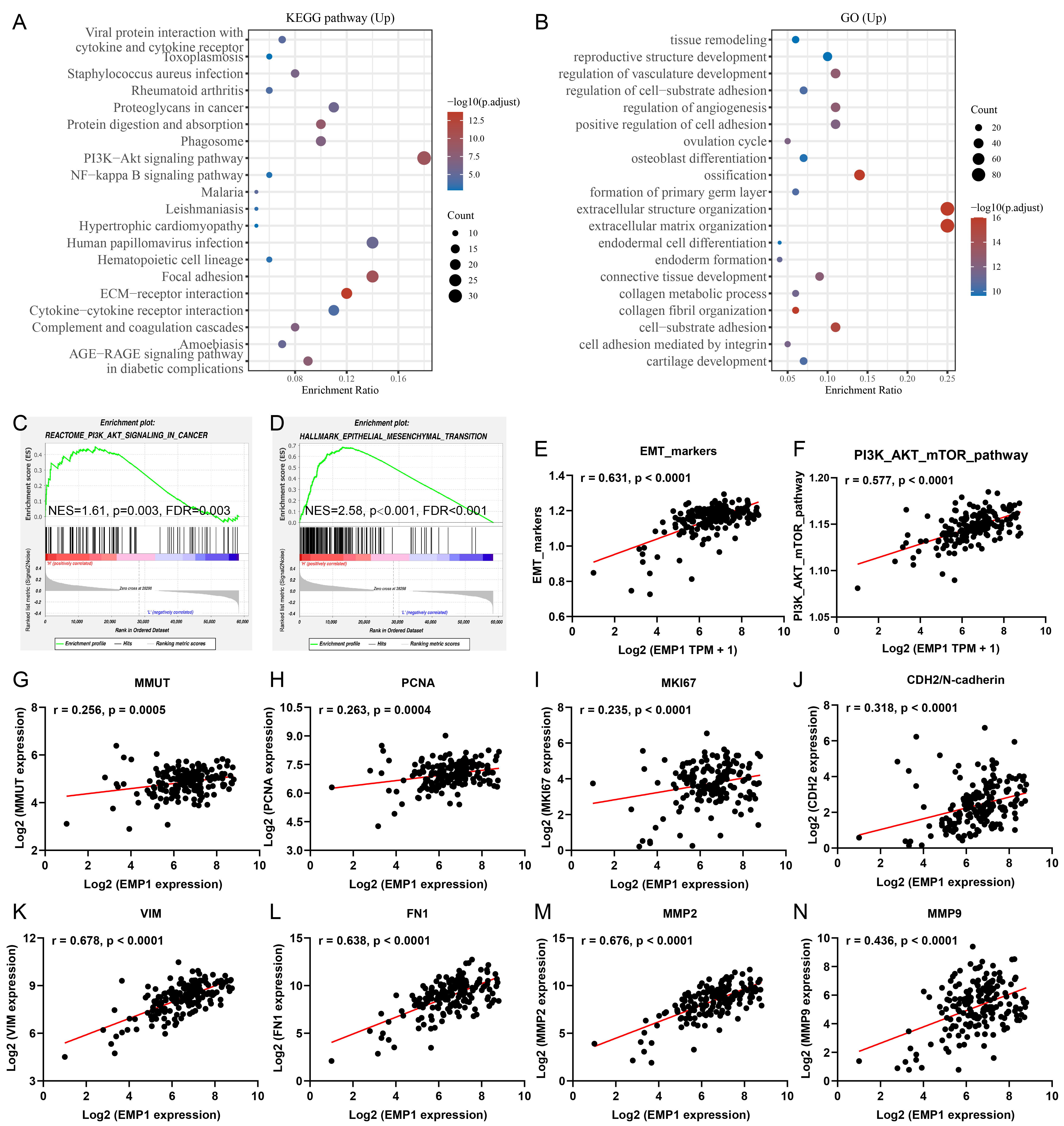


**Supplementary Figure 6. *EMP1* associates with EMT processes and the *PI3K/AKT* signaling cascade in PC cells.** (A-B) KEGG and GO enrichment analyses were conducted on differentially expressed genes between high and low EMP1 groups from the TCGA-PAAD dataset. (C-D) GSEA underscores a significant association of EMP1 mRNA levels with EMT and the *PI3K/AKT* signaling axis in PC. (E-F) Within the TCGA-PAAD dataset, *EMP1* exhibited consistent trends with EMT indicators and the PI3K/AKT signaling pathway. (G-N) Correlations were observed between *EMP1* and proliferation markers like *MMUT, PCNA,* and *MKI67*, as well as EMT-related markers such as *N-cadherin, VIM, FN1, MMP2,* and *MMP9* in the TCGA-PAAD dataset.


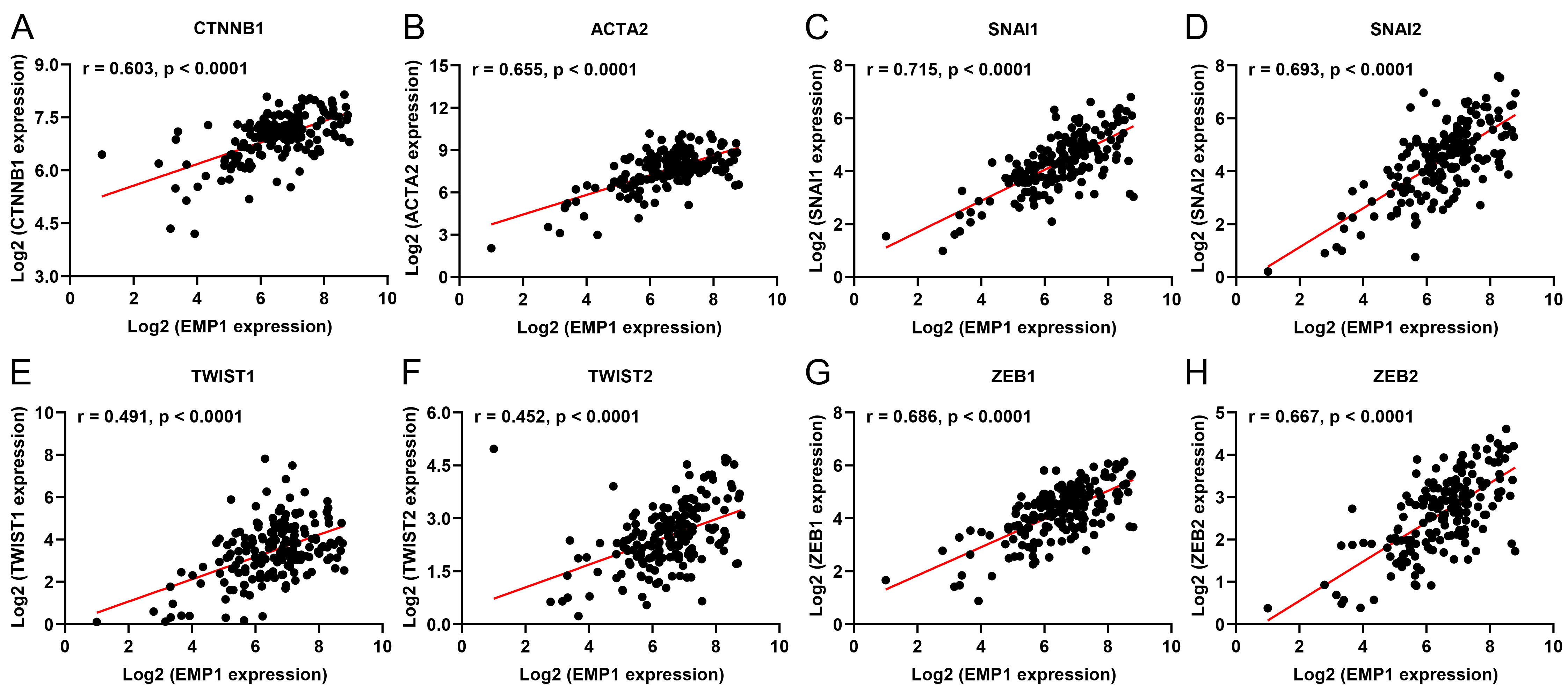


**Supplementary Figure 7. EMP1 exhibits a positively correlation with EMT in PC cells.** (A-H) Notable associations were identified between *EMP1* and EMT-related markers such as *CCNNB1, ACTA2, SNAI1, SNAI2, TWIST1, TWIST2, ZEB1,* and *ZEB2* within the TCGA-PAAD dataset.


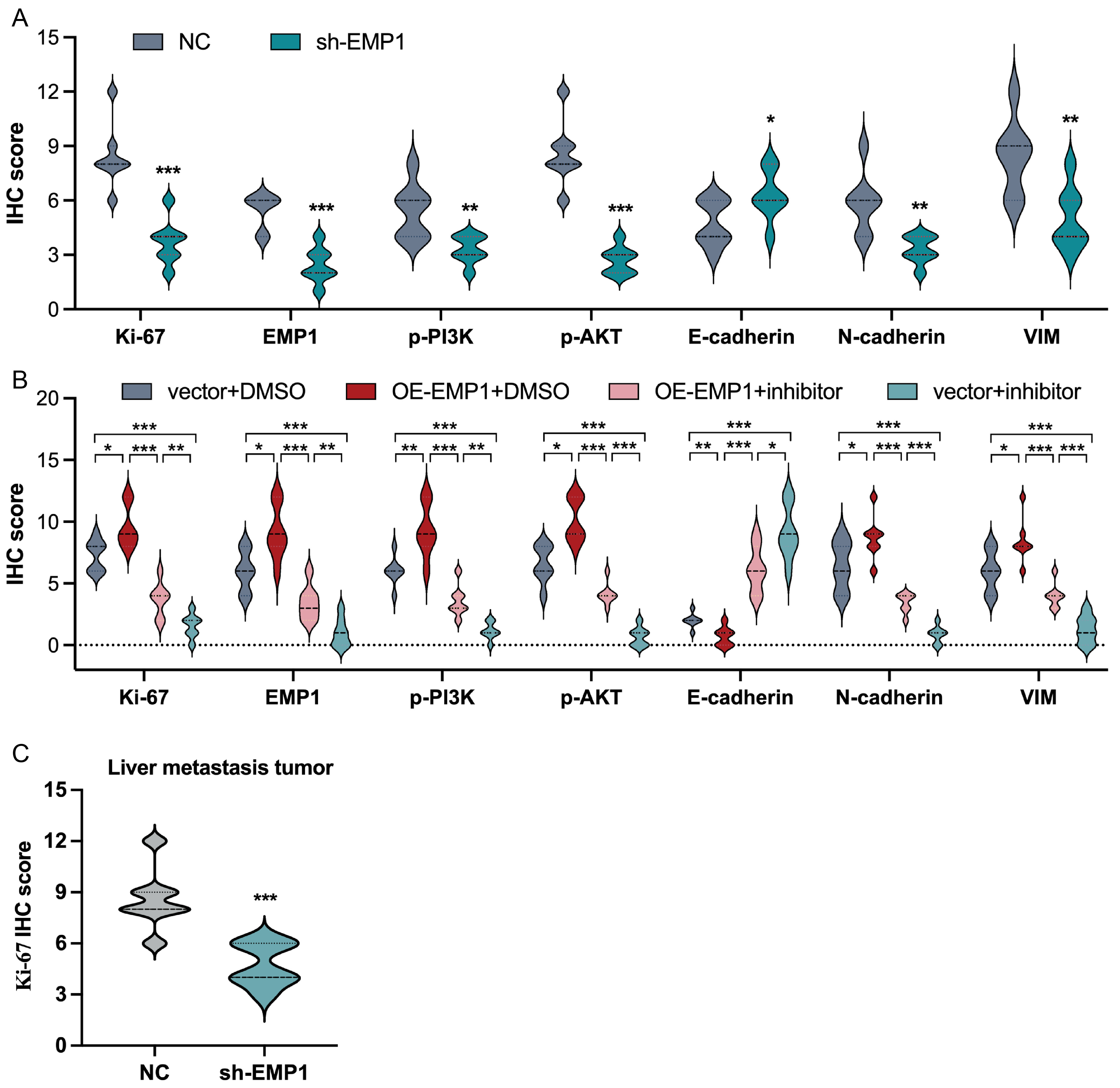


**Supplementary Figure 8. (**A-C) Quantitative immunohistochemical analysis of subcutaneous tumors from various groups for Ki-67, EMP1, p-PI3K, p-AKT, E-cadherin, N-cadherin, and Vimentin.


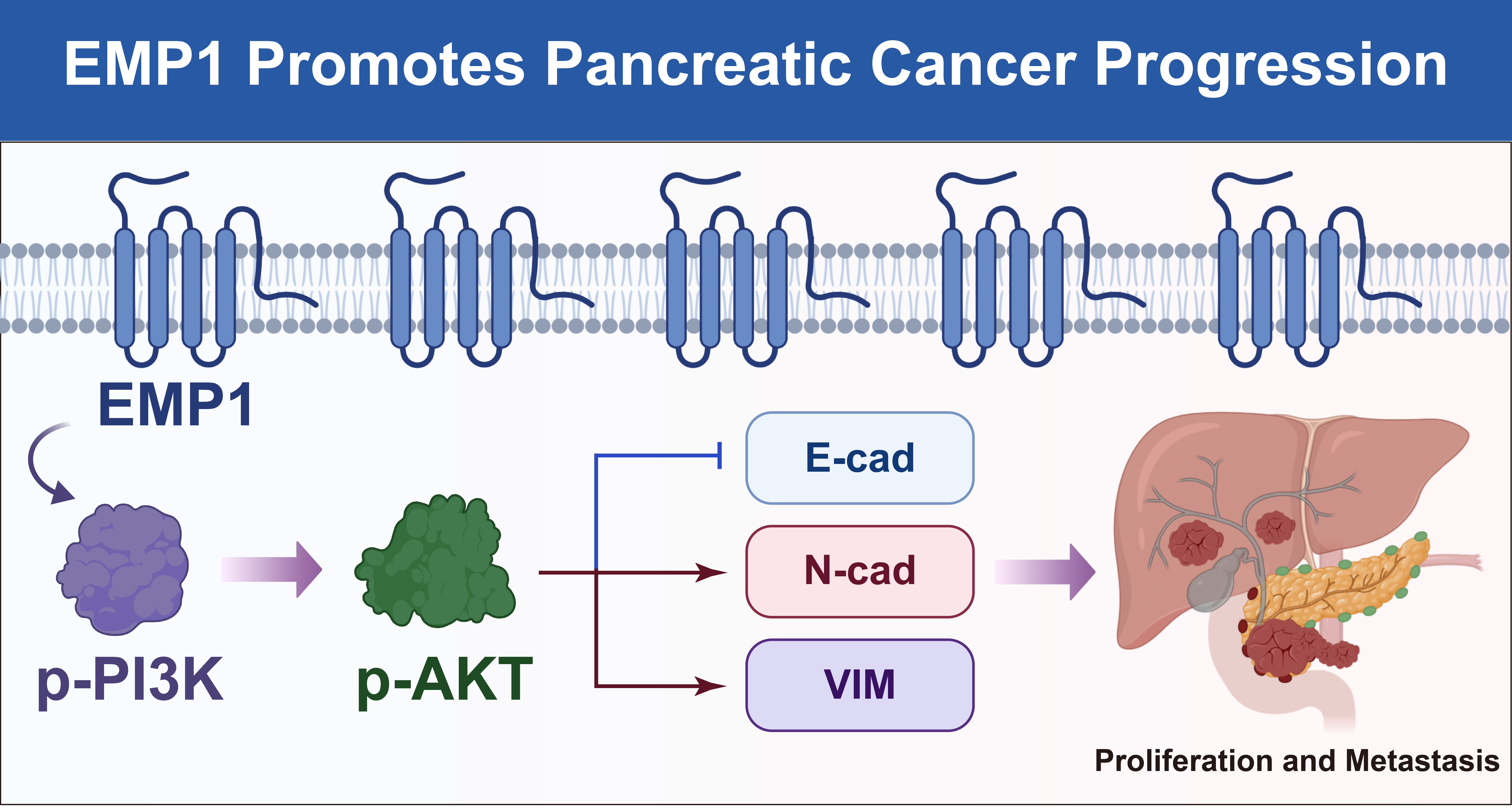


**Supplementary Figure 9. Schematic diagram.** EMP1 modulates PC progression via the PI3K/AKT signaling pathway.
