# Supplementary material for: The role of the aging process and related factor EMP1 in promoting progression of resectable pancreatic cancer
Source: Genes Dis. 2024 Dec 15;12(5):101490. doi: 10.1016/j.gendis.2024.101490 (PMC12221743; doi:10.1016/j.gendis.2024.101490)
Supplement: Multimedia component 2 [file mmc2.docx]

**Supplementary Tables**

| Supplementary Table 1. Clinical characteristics of pancreatic cancer stratified according to age in SEER pancreatic cancer patients | | | |  |
| --- | --- | --- | --- | --- |
| Characteristics | < 60 years (n = 5009) | >= 60 years (n = 13893) | p-value | |
| Gender |  |  |  | |
| female | 2349 (46.9%) | 6709 (48.3%) | 0.093 | |
| male | 2660 (53.1%) | 7184 (51.7%) |  | |
| stage_T |  |  |  | |
| T0 | 9 (0.2%) | 24 (0.2%) | <0.001 | |
| T1 | 619 (12.4%) | 1145 (8.2%) |  | |
| T2 | 1013 (20.2%) | 2664 (19.2%) |  | |
| T3 | 2636 (52.6%) | 8030 (57.8%) |  | |
| T4 | 732 (14.6%) | 2030 (14.6%) |  | |
| stage_N |  |  |  | |
| N0 | 2510 (50.1%) | 7195 (51.8%) | 0.043 | |
| N1 | 2499 (49.9%) | 6698 (48.2%) |  | |
| stage_M |  |  |  | |
| M0 | 3717 (74.2%) | 10434 (75.1%) | 0.217 | |
| M1 | 1292 (25.8%) | 3459 (24.9%) |  | |
| Differentiation |  |  |  | |
| Well | 1403 (28.0%) | 2614 (18.8%) | <0.001 | |
| Moderately | 1940 (38.7%) | 5715 (41.1%) |  | |
| Poorly | 1527 (30.5%) | 5230 (37.6%) |  | |
| Undifferentiated | 139 (2.8%) | 334 (2.4%) |  | |

| Supplementary Table 2. Cox univariable and multivariable analysis of  clinicopathological variables in SEER pancreatic cancer patients | | | | | | | | | |  |
| --- | --- | --- | --- | --- | --- | --- | --- | --- | --- | --- |
| Clinical factor | Univariable analysis | | | |  | | Multivariable analysis | | | |
|  | HR | 95%CI | p-value |  | | HR | | 95%CI | p-value |  |
| Age ( >= 60 vs. ＜ 60 years) | 1.603 | 1.533-1.675 | ＜0.001 |  | | 1.531 | | 1.464-1.601 | ＜0.001 |  |
| Gender (Male vs. Female) | 1.059 | 1.021-1.098 | 0.002 |  | | 1.041 | | 1.004-1.080 | 0.029 |  |
| T (vs. T0) |  |  |  |  | |  | |  |  |  |
| T1 | 0.089 | 0.061-0.129 | ＜0.001 |  | | 0.209 | | 0.143-0.305 | ＜0.001 |  |
| T2 | 0.309 | 0.214-0.446 | ＜0.001 |  | | 0.463 | | 0.321-0.669 | ＜0.001 |  |
| T3 | 0.321 | 0.223-0.463 | ＜0.001 |  | | 0.456 | | 0.316-0.658 | ＜0.001 |  |
| T4 | 0.584 | 0.405-0.842 | 0.004 |  | | 0.678 | | 0.470-0.978 | 0.038 |  |
| N (N1 vs. N0) | 1.171 | 1.129-1.215 | ＜0.001 |  | | - | | - | - |  |
| M (M1 vs. M0) | 2.841 | 2.732-2.955 | ＜0.001 |  | | 2.449 | | 2.35-2.553 | ＜0.001 |  |
| Differentiation (vs. Well differentiated) |  |  |  |  | |  | |  |  |  |
| Moderately differentiated | 2.426 | 2.283-2.578 | ＜0.001 |  | | 2.044 | | 1.921-2.176 | ＜0.001 |  |
| Poorly differentiated | 3.913 | 3.682-4.158 | ＜0.001 |  | | 2.961 | | 2.781-3.154 | ＜0.001 |  |
| Undifferentiated | 3.788 | 3.363-4.266 | ＜0.001 |  | | 2.709 | | 2.402-3.054 | ＜0.001 |  |

Supplementary Table 3. Clinical characteristics of pancreatic cancer stratified according to EMP1 expression.

| Characteristics | Low group (n = 50) | High group (n = 121) | p-value |
| --- | --- | --- | --- |
| Gender |  |  |  |
| female | 20 (40.0%) | 46 (38.0%) | 0.944 |
| male | 30 (60.0%) | 75 (62.0%) |  |
| Age (years) |  |  |  |
| < 60 | 28 (56.0%) | 54 (44.6%) | 0.236 |
| >= 60 | 22 (44.0%) | 67 (55.4%) |  |
| Tumor size (cm) |  |  |  |
| 0-2 | 10 (20.0%) | 23 (19.0%) | 0.897 |
| < 2, >= 4 | 32 (64.0%) | 75 (62.0%) |  |
| > 4 | 8 (16.0%) | 23 (19.0%) |  |
| Lymph node metastasis |  |  |  |
| Negative | 40 (80.0%) | 83 (68.6%) | 0.186 |
| Positive | 10 (20.0%) | 38 (31.4%) |  |
| Metastasis |  |  |  |
| Negative | 47 (94.0%) | 111 (91.7%) | 0.848 |
| Positive | 3 (6.0%) | 10 (8.3%) |  |
| Perineuronal invasion |  |  |  |
| Negative | 36 (72.0%) | 85 (70.2%) | 0.965 |
| Positive | 14 (28.0%) | 36 (29.8%) |  |
| Clinical stage |  |  |  |
| I-II | 46 (92.0%) | 99 (81.8%) | 0.146 |
| III-IV | 4 (8.0%) | 22 (18.2%) |  |
| Differentiation |  |  |  |
| I-II | 44 (88.0%) | 81 (66.9%) | 0.008 |
| III-IV | 6 (12.0%) | 40 (33.1%) |  |

Supplementary Table 4. Cox univariable and multivariable analysis of clinicopathological variables and

EMP1 expression in relation to OS in pancreatic cancer patients.

| Clinical factor | Univariable analysis | | |  | Multivariable analysis | | |
| --- | --- | --- | --- | --- | --- | --- | --- |
|  | HR | 95%CI | p-value |  | HR | 95%CI | p-value |
| Gender (male vs. female) | 0.971 | 0.654-1.443 | 0.885 |  |  |  |  |
| Age ( >=60 vs. <60 years) | 1.113 | 0.757-1.637 | 0.585 |  |  |  |  |
| Tumor size  (vs. 0-2 cm) |  |  | 0.164 |  |  |  |  |
| < 2, >= 4 | 1.612 | 0.906-2.869 | 0.105 |  |  |  |  |
| > 4 | 1.879 | 0.964-3.661 | 0.064 |  |  |  |  |
| Lymph node metastasis (Positive vs. Negative) | 1.875 | 1.221-2.879 | 0.004 |  | 1.752 | 1.139-2.695 | 0.011 |
| Metastasis (Positive vs. Negative) | 1.347 | 0.723-2.51 | 0.348 |  |  |  |  |
| Perineuronal invasion  (Positive vs. Negative) | 0.688 | 0.425-1.115 | 0.129 |  |  |  |  |
| Clinical stage  (III-IV vs. I-II) | 1.579 | 0.972-2.565 | 0.065 |  |  |  |  |
| Differentiation (III-IV vs. I-II) | 1.583 | 1.045-2.396 | 0.030 |  | - | - | - |
| EMP1 expression (High vs. Low) | 4.485 | 2.718-7.401 | ＜0.001 |  | 4.401 | 2.661-7.281 | ＜0.001 |
| OS, overall survival; HR, hazard ratio | | |  |  |  |  |  |
